# Interaction Mechanism of Bovine Myosin Oxidation Inhibition by Epicatechin Gallate: Insights from Molecular Docking and Thermodynamic Analysis

**DOI:** 10.3390/foods15183332

**Published:** 2026-09-20

**Authors:** Xin Li, Meijuan Xu, Min Li, Xinming Yin, Huang Zhang, Xueqin Gao, Changbin Li, Zhida Sun, Bijun Xie, Jian Zou

**Affiliations:** 1 College of Food Science, Henan University of Animal Husbandry and Economy, Zhengzhou 450046, China; lixinxin_619@126.com (X.L.); xumeijuan113@163.com (M.X.); 261016@hnuahe.edu.cn (M.L.); xinmingyin@hotmail.com (X.Y.); 81870@hnuahe.edu.cn (H.Z.); kfgxq03@163.com (X.G.); lichangbin911@163.com (C.L.); 2 College of Food Science and Technology, Huazhong Agricultural University, Wuhan 430070, China; sunzhida@mail.hzau.edu.cn (Z.S.); bijunxie@sina.com (B.X.)

**Keywords:** bovine myosin, epicatechin gallate, dose–effect relationship, inhibition of protein oxidation, molecular docking, isothermal titration calorimetry, biphasic dose–response relationship

## Abstract

Protein oxidation is a critical factor governing meat quality. Although lotus seedpod proanthocyanidins (LSPC) can inhibit protein oxidation in beef during refrigerated storage, the underlying mechanism remains unclear. This study investigated the antioxidant activity and conformation impacts of epigallocatechin gallate (ECG), the principal active constituent of LSPC, on myosin from the perspective of molecular interactions. Isothermal titration calorimetry (ITC), molecular docking, and multispectral analyses were applied to characterize ECG–myosin interactions and unravel the mechanism underlying myosin oxidation inhibition. The results showed that ECG exerted a biphasic, dose-dependent effect on myosin conformation and related functional properties. At 10–30 μmol/L, ECG significantly enhanced the radical scavenging capacity of myosin and alleviated oxidative damage to amino acid side chains. Mechanistically, ECG bound spontaneously to myosin via entropy-driven hydrogen bond and hydrophobic interactions, inducing conformational rearrangement characterized by an α-helix-to-β-sheet transition, reduced surface hydrophobicity, and stabilization of secondary and tertiary structures, thereby inhibiting oxidative denaturation of myosin. However, at 40 μmol/L, ECG still retained antioxidant capacity toward myosin, while it increased surface hydrophobicity and reduced fluorescence intensity. These changes further aggravated the structural disorder, led to protein aggregation and microstructure damage, and ultimately caused the instability of myosin. These findings underscore the necessity of optimizing ECG dosage for reliable application in meat processing and provide experimental evidence supporting ECG and LSPC as natural antioxidants for meat systems.

## 1. Introduction

Beef is a rich source of dietary protein and serves as a fundamental provider of high-quality nutrients for human consumption. Nevertheless, following slaughter, the endogenous antioxidant system of beef undergoes degradation. Concurrently, triggered by extrinsic factors including light exposure and oxygenation, protein oxidation persists throughout the entire refrigerated distribution and retail cycle. This process induces the depletion of sulfhydryl groups, the generation of dityrosine, and ultimately leads to the cross-linking and aggregation of protein molecules [1,2,3]. Consequently, this contributes to quality degradation phenomena of beef such as meat discoloration, drip loss, textural toughening, and the depletion of essential amino acids [4], thereby significantly diminishing consumer purchasing intent.

As the major component of meat protein, myofibrillar proteins account for approximately 55% of total muscle protein and govern the textural properties of meat products [5]. Myosin is the most abundant protein in beef myofibrils. As a Y-shaped biomacromolecule rich in sulfhydryl groups, myosin is susceptible to oxidation, where sulfhydryl groups convert into disulfide bonds and further mediate inter-protein cross-linking [6,7]. Previous studies have demonstrated that the functional performance of myosin is closely correlated with its redox state, molecular-weight distribution, and spatial conformational stability; these properties directly determine the final quality of meat and meat products during refrigerated storage [8,9]. Accordingly, inhibiting myosin oxidation and preserving conformational stability of myosin represents a vital strategy for maintaining the quality of chilled beef under refrigeration.

Over the last several years, synthetic antioxidants have been widely used in food processing owing to their high efficiency and stable performance. Nevertheless, their potential health risks have raised public concerns and restricted their application, thereby driving the exploration of natural plant antioxidant alternatives. Numerous studies have verified that typical polyphenols, including grape seed proanthocyanidins, tea polyphenols, guava leaf polyphenol, and mulberry polyphenols, can effectively inhibit meat protein oxidation and improve meat quality [9,10,11,12,13]. However, the high cost of raw materials greatly restricts their large-scale industrial application. Fortunately, Lotus seedpod proanthocyanidins (LSPC), extracted from abandoned lotus seedpod by-products and mainly composed of catechins, epigallocatechin, and ECG, exhibit prominent cost advantages and excellent antioxidant and antibacterial properties [14,15]. Existing research has confirmed that LSPC can inhibit protein oxidation in chilled beef and extend its shelf life by 4 to 8 days during refrigerated storage [16].

Recent studies have demonstrated that covalent and non-covalent interactions between plant polyphenols and proteins can endow protein complexes with antioxidant capacity, optimize protein functional properties, regulate surface hydrophobicity and structural stability, and thereby confer nutritional benefits to meat products [17,18]. For example, enhanced antioxidant activity has been observed for non-covalent complexes formed by chlorogenic acid-casein and epigallocatechin gallate-β-lactoglobulin [19,20]. In addition, interactions between grape-seed proanthocyanidins and silver carp myofibrillar protein suppress protein oxidation and enhance the water-holding capacity and thermal stability of silver carp myofibrillar protein [21]. Notably, emerging studies have further explored the interaction mechanism between monomeric polyphenols and meat myosin. Guo et al. [22]. confirmed that hydrophobic interaction and hydrogen bonding are the predominant binding forces between *Coregonus peled* myofibrillar protein and polyphenols, including chlorogenic acid (CA), ECG, and epigallocatechin gallate (EGCG), via multi-spectral and molecular docking analysis. Nevertheless, the effects of these polyphenols on the oxidative stability of myofibrillar protein have not been systematically studied. Zhang et al. [23]. revealed that tea phenolic compounds, including ECG, improve the oxidative stability of myosin and modulate its surface hydrophobicity during the thermal processing of tea-stewed beef, and explored the underlying interaction mechanisms via molecular docking and molecular dynamics simulations. However, most previous studies have focused primarily on thermal processing. In particular, the mechanism by which LSPC and its major components inhibit beef protein oxidation and modulate protein conformation through polyphenol–protein molecular interactions during low-temperature chilled storage remains unclear and unreported.

As LSPC is a complex mixture containing multiple active ingredients, elucidating its precise molecular interactions with proteins remains challenging, thereby limiting the in-depth understanding of its antioxidant mechanism. Considering the compositional complexity of beef proteins, this study selected ECG—one of the major active components of LSPC—and bovine myosin as research objects. By integrating Fourier transform infrared spectroscopy (FTIR), thermodynamic analysis, and molecular docking, this study investigated the dominant binding forces, binding modes, and binding sites between ECG and myosin, and clarified the underlying mechanism by which ECG inhibits myosin oxidation and stabilizes protein conformation. This work not only extends the theoretical framework of polyphenol–meat protein interactions but also provides fundamental references and experimental support for both the exploration of ECG and other LSPC components’ regulatory effects on protein oxidation and their prospective application.

## 2. Materials and Methods

### 2.1. Materials

Due to the complex structure of LSPC (Figure S1 in Supplementary Materials), it is difficult to conduct comprehensive and in-depth research on its intermolecular interactions with proteins. Therefore, ECG, one of the major components of LSPC, was selected as the research object in this study (The detailed screening procedure for ECG is presented in Part 2 of the Supplementary Materials). ECG (purity > 98%) was purchased from Chengdu Purifa Biological Co., Ltd. (Chengdu, China).

Chilled beef (*longissimus dorsi*) was purchased from Henan Yisai Beef Co., Ltd. in Jiaozuo (Jiaozuo, China) and transported to the laboratory via rapid cold chain transportation at 4 °C and used for the experiment.

### 2.2. Chemicals and Reagents

2,2-Diphenyl-1-picrylhydrazyl (DPPH), β-mercaptoethanol, 2,2′-amino-bis(3-ethylbenzothiazol-6-sulfonic acid) diammonium salt (ABTS), Tris (hydroxymethyl) aminomethane, 2, 4, 6-tripyridinyltriazine, Adenosine triphosphate (ATP), O-phthalaldehyde (OPA), Maleic acid, diethyl (α-aminoethyl ether) tetraacetic acid (EGTA) were purchased from Shanghai Maclean Technology Co., Ltd. (Shanghai, China). 1-anilino naphthalene-8-sulfonic acid (ANS) was purchased from Sigma Aldrich (Shanghai) Trading Co., Ltd. (Shanghai, China). Sulfhydryl Assay Kit was purchased from Nanjing Jiancheng Bio Co., Ltd. (Nanjing, China).

### 2.3. Extraction of Myosin from Chilled Beef

Myosin was extracted from chilled beef according to previously reported methods with appropriate modifications [24]. The *longissimus dorsi* muscle of chilled beef was minced into fine meat particles. Subsequently, a total of 50.00 g of the minced meat was mixed with 10.00 volumes of Reagent A (0.1 mol/L KCl, 20 mmol/L Tris-HCl buffer, pH adjusted to 7.5 with HCl), homogenized at 4 °C (10,000× *g*, 1 min), and incubated at 4 °C for 15 min, followed by centrifugation at 4 °C (8000× *g*, 10 min). The resulting precipitate was resuspended in 5.00 volumes of Reagent B (0.45 mol/L KCl, 5 mmol/L β-mercaptoethanol, 0.2 mol/L magnesium acetate, 1 mmol/L EGTA, 20 mmol/L Tris-HCl buffer, pH adjusted to 6.8 with maleic acid), and ATP was added to a final concentration of 10 mmol/L. The mixture was incubated at 4 °C for 90 min and then centrifuged at 4 °C (10,000× *g*, 10 min). The resulting supernatant was slowly diluted with 5 volumes of 1 mmol/L KHCO_3_ solution, incubated at 4 °C for 20 min, and centrifuged at 4 °C (11,000× *g*, 10 min). The precipitate was resuspended in 2.5 volumes of Reagent C (0.50 mol/L KCl, 5.00 mmol/L β-mercaptoethanol, 20 mmol/L Tris-HCl buffer, pH adjusted to 7.5 with HCl) and incubated at 4 °C for 10 min. Then, 7.00 volumes of 1 mmol/L KHCO_3_ solution were added slowly, accompanied by the addition of MgCl_2_ to a final concentration of 10 mmol/L. The mixture was allowed to stand at 4 °C for 12 h, followed by centrifugation at 4 °C (10,000× *g*, 20 min), and the final precipitate was dissolved by adding 20 mmol/L Tris-HCl buffer (pH 7.5, containing 0.50 mol/L NaCl). The myosin content of the final precipitate solution was measured within 48 h according to the method described by Gornall with bovine serum albumin (BSA) used as the standard [25], and the final myosin concentration then was adjusted using 20 mmol/L Tris-HCl buffer for sample preparation.

### 2.4. Sample Preparation

ECG aqueous solutions were prepared at concentrations of 20, 40, 60, and 80 μmol/L, respectively. Each ECG solution (10 mL) was fully mixed with an equal volume of 1 mg/mL myosin solution. Distilled water replaced the ECG solution in the blank control group. The final myosin concentration in all experimental and control samples was maintained at 0.5 mg/mL in all samples, and the corresponding final ECG concentrations in the reaction systems were 0, 10, 20, 30, and 40 μmol/L, respectively. ECG solutions without myosin were prepared as independent ECG control groups. All samples were incubated at 4 °C for 12 h to form ECG–myosin mixtures with different ECG concentrations. Subsequently, the ECG–myosin mixtures were transferred into regenerated cellulose dialysis tubing (molecular weight cutoff = 3500 Da) and dialyzed at 4 °C with gentle magnetic stirring against 50.00–100.00 volumes of buffer solution. The dialysate was renewed at intervals of 2–4 h and 6–8 h, followed by continuous overnight dialysis for a total of 24 h to completely remove unbound free ECG.

### 2.5. Effects of ECG on the Antioxidant Capacity of Myosin

#### 2.5.1. Determination of Oxidative Stability of the ECG-Myosin Mixture

The oxidative stability of ECG–myosin mixtures was evaluated via electron paramagnetic resonance (EPR) spectrometer (EMXPlus, Bruker, Ettlingen, Germany) according to a previously reported method by Jongberg et al. [26]. Briefly, the samples prepared in Section 2.4 were pre-frozen at −80 °C for 24 h, spread evenly on metal trays, and freeze-dried using a lyophilizer (Lyobeta 25, Telstar Industrial, Barcelona, Spain). Freeze-drying was performed under a vacuum pressure of 5 Pa at −80 °C for 48 h. After lyophilization, the dried samples were ground into powder at 4 °C. An aqueous sample suspension (1.00 mg/mL) was prepared and dispersed by homogenization to ensure identical mass of myosin-containing sample in each aliquot for EPR measurement. To detect hydroxyl radicals, 5,5-dimethyl-1-pyrroline N-oxide (DMPO) (Sigma-Aldrich, Darmstadt, Germany) was used as a spin trap. A 100.00 mmol/L DMPO working solution was prepared, and 100.00 μL of sample suspension was mixed with an equal volume of DMPO solution. After homogenization, the mixture was loaded into a capillary tube and flame-sealed. The capillary sample was placed inside the EPR cavity for measurement. All EPR spectra were collected under dark conditions. The instrumental parameters were set as follows: microwave power, 4 mW; central field, 336 mT; scan width, 5 mT; scan time, 2 min; modulation width, 0.125 mT; amplitude, 200; time constant, 0.3 s; and number of scans, 1. After spectral acquisition, spectral processing and normalization were performed using Bruker Xenon 1.1b60 software. The peak area of DMPO–OH adduct was calculated, and all EPR signals were normalized to the mass of myosin in each capillary sample.

#### 2.5.2. Determination of Antioxidant Capacity of the ECG–Myosin Mixtures

The determination of DPPH free radical scavenging was based on the method of Bellucci et al. [27]; 2.00 mL of each sample solution, with a uniform myosin concentration of 0.50 mg/mL across all groups except the ECG control group was pipetted, 0.20 mmol/L DPPH solution was added in equal volume, shaken and reacted in the dark for 30 min.

The DPPH radical scavenging activity was calculated using the formula shown below.

$$DPPH\;free\;radical\;scavenging\;rate\;(\%)=\left(1-\frac{A_{1}-A_{2}}{A_{0}}\right)\times 100\%$$

where: *A*_1_ is the absorbance at 517 nm after the reaction of the sample with DPPH solution; *A*_2_ is the absorbance of the sample and an equal volume of ethanol at 517 nm; *A*_0_ is the absorbance of DPPH solution with an equal volume of ethanol at 517 nm.

The determination of hydroxyl radicals (•OH) scavenging capacity was followed by Smirnoff et al. [28]. Briefly, 2.00 mL of sample solution, 2.00 mL of 6.00 mmol/L FeSO_4_ solution, and 2.00 mL of 6.00 mmol/L H_2_O_2_ solution were sequentially mixed and incubated for 10 min, followed by the addition of 2.00 mL of 6.00 mmol/L salicylic acid solution. After homogenization and 30 min of dark reaction, the absorbance (*D*_i_) at 510 nm was determined using an UV–visible spectrophotometer. The background optical density of the sample (*D*_j_) was measured by replacing salicylic acid solution with distilled water, while the blank control optical density (*D*_0_) was obtained by substituting the sample solution with distilled water. The hydroxyl radical scavenging rate was calculated using the following formula:

$$Hydroxyl\;radical\;scavenging\;rate\;(100\%)=\frac{D_{i}-D_{j}}{D_{0}}\times 100\%$$

The determination of ABTS radical cation (ABTS•^+^) scavenging capacity was assessed based on the method of Almajano et al. [29]. In brief, the ABTS stock solution was prepared by mixing 7 mmol/L ABTS and 2.45 mmol/L potassium persulfate in PBS (pH 7.4) and was used within 24 h. Before detection, the stock solution was diluted 1:100 with PBS to obtain a working solution with an absorbance of 0.70 ± 0.02 at 734 nm. A total of 20.00 μL of sample solution was mixed with 2.00 mL of ABTS working solution, and PBS was used as the blank control. The absorbance at 734 nm was recorded every minute for 7 min. The ABTS•^+^ scavenging rate was calculated based on the measured absorbance values. The absorbance variation of the sample (Δ*A*_sample_) was calculated according to the following formula:

$$\Delta A_{\mathrm{sample}}=\frac{A_{1}-A_{2}\;}{A_{1}}-\frac{A_{3}-A_{4}\;}{A_{3}}$$

where: *A*_1_ represents the absorbance of the sample group at 0 min, *A*_2_ represents the absorbance of the sample group at 7 min, *A*_3_ represents the absorbance of the blank group at 0 min, and *A*_4_ represents the absorbance of the blank group at 7 min.

### 2.6. Effects of ECG on Myosin Amino Acid Side Chains Oxidation

#### 2.6.1. Determination of Myosin Sulfhydryl Group Content

The determination of sulfhydryl groups content was carried out in accordance with the operating instructions of the Sulfhydryl Assay Kit.

#### 2.6.2. Determination of Free Amino Group Content

The determination of free amino groups content referred to the OPA method of Lv et al. [30]. Reagent 1 was prepared as follows: 40.00 mg of OPA was accurately weighed and dissolved in 1 mL of methanol, followed by the addition of 3.00 mL of distilled water. The mixture was blended thoroughly and stored in a brown reagent bottle for subsequent use. Reagent 2 was prepared by mixing 2.50 mL of 20% (*w*/*w*) SDS solution, 25 mL of 0.1 mol/L borax solution and 100.00 µL of β-mercaptoethanol, then the mixture was diluted to 50 mL with distilled water and shaken uniformly. During the determination process, 0.3 mL of Reagent 1 and 3.70 mL of Reagent 2 were added into test tubes and mixed adequately, followed by the addition of 2.00 mL of ECG-myosin mixture sample solution. After thorough blending, the reaction system was incubated in a 35 °C water bath for 2 min. The UV-Vis absorbance of the reaction solution was recorded at 340 nm. Meanwhile, a control group was prepared by replacing the sample solution with 2.00 mL of distilled water. A standard curve was established using lysine standard solution instead of the sample solution, to quantify the free amino group content in the tested samples.

#### 2.6.3. Determination of Dityrosine Level

The dityrosine level was determined according to the method described by Zhang et al. [31]. In brief, 4.00 mL of each sample was centrifuged at 5000× *g* for 10 min at 4 °C. The fluorescence intensity of the supernatant was measured using a fluorescence spectrophotometer (F-4600, Foss, Hillerød, Denmark) with an excitation wavelength of 325 nm, an emission wavelength of 420 nm, and a slit width of 10 nm. The myosin concentration of the supernatant was quantified via the Coomassie brilliant blue method. The dityrosine level was defined as the corrected fluorescence intensity, which was calculated by normalizing the raw fluorescence intensity to the corresponding myosin concentration. All results were expressed in arbitrary units (A.U.) [32].

### 2.7. Determination of Secondary Structural Changes in ECG–Myosin Mixture

#### 2.7.1. Circular Dichroism (CD) Spectroscopy

The sample solution was diluted with phosphate-buffered saline (PBS) to yield a myosin concentration of 0.20 mg/mL. A 200.00 μL aliquot of diluted sample was subjected to circular dichroism measurement using a CD spectropolarimeter (J-1500, Jasco, Tokyo, Japan) under continuous nitrogen purge in the far-UV region (200–250 nm). A quartz cuvette with an optical path length of 0.1 cm was employed for data acquisition. Ellipticity was collected at the scan speed of 100 nm/min, 3 accumulations, 1 nm bandwidth, 0.25 s response time and 0.5 nm resolutions. Buffer blanks were subtracted from all sample spectra. The CD signals were converted into mean residue ellipticity (MRE, degree·cm^2^·dmol^−1^) according to Yang’s method [33]. Secondary-structure fractions (α-helix, β-sheet, β-turn, and random coil) were calculated from the MRE spectra using the DichroWeb web-server (Birkbeck, University of London, London, UK). The NRMSD was used to assess fitting quality. The CONTINLL algorithm combined with the SMP180 reference dataset was selected for spectral deconvolution.

#### 2.7.2. FTIR Spectroscopy

The samples were frozen according to the freeze-drying procedure described in Section 2.5.1; 100.00 mg of each freeze-dried sample was mixed separately with potassium bromide (KBr) in a 1:100 ratio and ground into a homogeneous fine powder, which was then compressed into a transparent thin sheet. The sheet was scanned using a Fourier transform infrared spectrometer (Nexus 470, Thermo Fisher, Waltham, MA, USA) in the wavenumber range of 4000–400 cm^−1^ at a resolution of 8 cm^−1^. FTIR spectra were recorded as the average of 32 cumulative scans.

### 2.8. Determination of Tertiary Structure Changes in ECG–Myosin Mixture

#### 2.8.1. Intrinsic Tryptophan Fluorescence

Intrinsic tryptophan fluorescence was measured according to the method described by Jiang et al. [34]. Fluorescence emission spectra of sample solutions containing myosin at 0.5 mg/L were collected using an F-4600 fluorescence spectrophotometer (Hitachi, Tokyo, Japan) at an excitation wavelength of 280 nm. Spectra were recorded over the emission range of 300–450 nm. Both excitation and emission slit widths were set to 5 nm, and the data acquisition rate was 240 nm/min. All spectra were corrected for the inner-filter effect caused by ECG.

#### 2.8.2. Surface Hydrophobicity

Surface hydrophobicity was determined using the ANS fluorescence probe method described by Huang et al. [35]. Sample solutions were adjusted to gradient myosin concentrations of 0.00 mg/mL, 0.05 mg/mL, 0.10 mg/mL, 0.15 mg/mL, and 0.20 mg/mL, respectively. Then, 10 μL of 8 × 10^−3^ mol/L ANS solution prepared in 0.1 mol/L phosphate buffer (pH 7.4) was added to 2 mL of each diluted sample. After incubation in the dark for 10 min, the fluorescence intensity was measured using a fluorescence spectrophotometer (Hitachi, Tokyo, Japan), with the excitation wavelength set at 390 nm, emission wavelength at 479 nm, and slit width of 5 nm. The surface hydrophobicity was calculated as the initial slope of the fluorescence intensity versus protein concentration plot via linear regression analysis.

### 2.9. Determination of Microstructure Changes in ECG–Myosin Mixture

Samples were frozen-dried according to the freeze-drying procedure described in Section 2.5.1 without powder grinding. The freeze-dried samples were cut into small pieces (≤3 mm thickness, approximately 15.00–20.00 mg) and fixed in 2.5% glutaraldehyde (prepared in 0.10 mol/L phosphate buffer, pH 7.2) at a fixative-to-sample volume ratio of 10:1 for 4 h at 4 °C. Subsequently, the fixed samples were rinsed three times with 0.10 mol/L phosphate buffer, with each rinsing lasting 10–15 min. The specimens were subsequently transferred to a critical-point dryer and immersed in 100% isoamyl acetate for solvent exchange, until the residual buffer solution was completely replaced by isoamyl acetate. The samples were further treated with liquid CO_2_ to fully replace isoamyl acetate. Afterwards, the temperature and pressure were elevated above the critical point of CO_2_, and supercritical CO_2_ was slowly vented to achieve complete sample drying. The dried protein blocks were sputter-coated with gold for subsequent scanning electron microscopy (SEM, SU8010, Tianmei, Shanghai, China) observation. The coated samples were observed and imaged under 3000× magnification with an acceleration voltage of 15 kV using a field-emission scanning electron microscopy.

### 2.10. ITC Measurement

The interaction type and binding constant between ECG and myosin were determined via isothermal titration calorimetry (Nano-ITC, TA Instruments, New Castle, DE, USA). Specifically, the ECG aqueous solution and myosin (molecular weight 480 kDa) solution were adjusted to 400.00 μmol/L and 20.00 μmol/L, respectively, using 20.00 mmol/L Tris-HCl buffer. All samples were vacuum-degassed at 4 °C for 15 min with gentle stirring using a Degassing Station (TA Instruments, New Castle, DE, USA) prior to loading. For titration measurements, 200.00 μL of 20.00 μmol/L myosin solution was loaded into the ITC sample cell, and 50.00 μL of 400.00 μmol/L ECG solution was filled in the titration syringe. The ECG solution was titrated into the myosin solution via 20 successive injections (2.50 μL per injection) with an interval of 180 s between each injection. The sample cell was stirred continuously at 200 r/min throughout the titration process. For the blank control, ECG solution was titrated into pure 20.00 mmol/L Tris-HCl buffer (pH 7.5, containing 0.50 mol/L NaCl) under identical conditions. All experiments were performed in three independent replicates. The data from the first injection were discarded, and the background heat signals from buffer titration were subtracted for all thermograms.

All corrected titration data were analyzed using Nano Analyze 2021 software (TA Instruments, New Castle, DE, USA) and nonlinearly fitted with a single-site binding model to obtain fundamental binding parameters, including the binding constant (*Ka*), binding stoichiometry (n), and enthalpy change (Δ*H*). The corresponding Gibbs free energy change (Δ*G*) and entropy change (Δ*S*) were calculated according to the thermodynamic equation Δ*G =* −*RT* ln *Ka* and Δ*G =* Δ*H* − *T*Δ*S.* The fitting quality of each titration curve was evaluated based on the residual sum of squares (RSS). The parameters uncertainties were derived from bootstrap resampling statistics implemented in Nano Analyze 2021software. Furthermore, the 95% confidence intervals (95% CI) of all fitted thermodynamic parameters were calculated to verify the statistical reliability and goodness-of-fit of the binding model.

### 2.11. Analysis of the Interaction Force Between ECG and Myosin

The interaction forces between ECG and myosin were determined according to the method reported by Zhang et al. [36] with minor modifications. Briefly, following the sample preparation procedure described in Section 2.4, ECG and myosin concentrations were increased proportionally to prepare the ECG–myosin mixture and control group samples with a final myosin concentration of 10.00 mg/mL; 1.00 mL of the samples were mixed thoroughly with 5.00 mL of five different reaction solutions: Solution A (0.05 mol/L NaCl), Solution B (0.60 mol/L NaCl), Solution C (0.60 mol/L NaCl containing 1.50 mol/L urea), Solution D (0.60 mol/L NaCl containing 8.00 mol/L urea), and Solution E (0.60 mol/L NaCl containing 8 mol/L urea and 0.05 mol/L β-mercaptoethanol). The mixtures were homogenized at 7000 r/min for 30 s and centrifuged at 10,000 r/min for 5 min at 4 °C. The protein concentration in the supernatant was quantified using the Coomassie brilliant blue method. The different types of intermolecular interaction forces were calculated as follows:

Ionic bond (electrostatic interaction) = Protein content in Solution B − Protein content in Solution A

Hydrogen bond = Protein content in Solution C − Protein content in Solution B

Hydrophobic interaction = Protein content in Solution D − Protein content in Solution C

Disulfide bond = Protein content in Solution E − Protein content in Solution D

### 2.12. Molecular Docking

**Figure 1:**
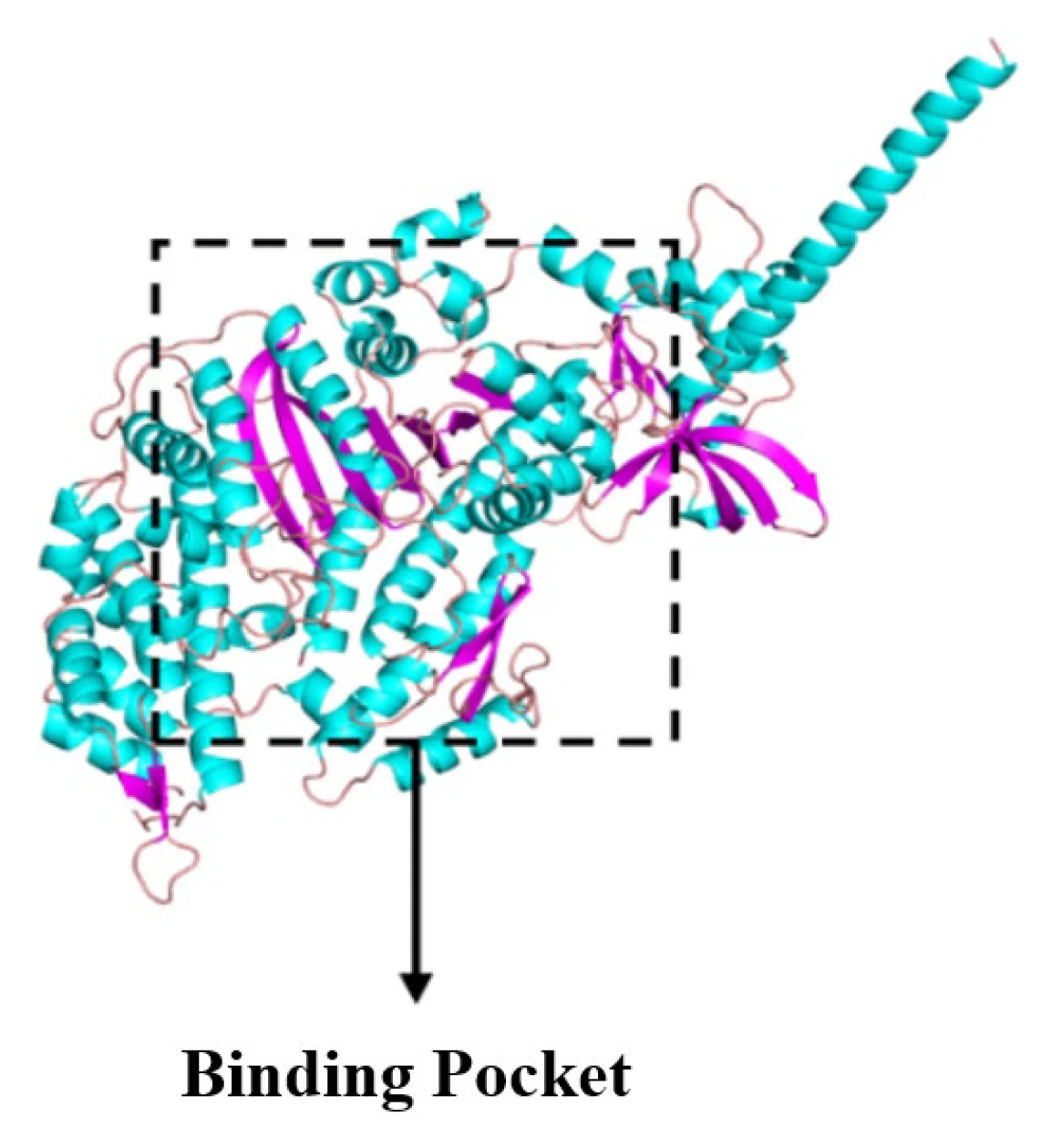
Myosin structure and key active pocket.

**Figure 2:**
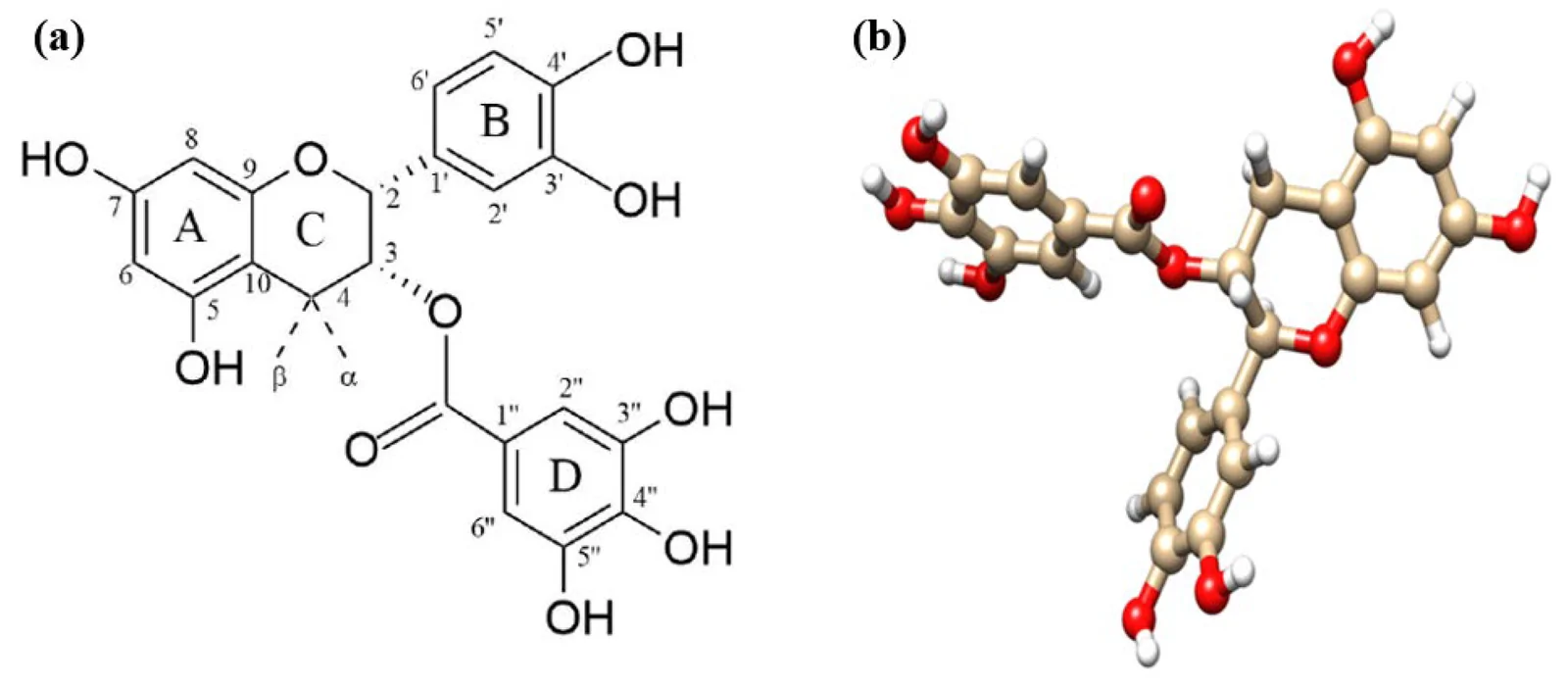
Chemical structure of ECG. (**a**) 2D chemical formula; (**b**) simulated three-dimensional molecular structure.

Homology modeling of bovine myosin heavy chain was performed on the SWISS-MODEL server (https://swissmodel.expasy.org/). The PDB entry 6FSA was selected as the optimal template, which shared 78.10% sequence identity and 97.31% alignment coverage with bovine myosin heavy chain to support reliable sequence alignment. The resulting homology-modeled myosin structure was pretreated with AutoDock Tools 1.5.6 (The Scripps Research Institute, San Diego, CA, USA) to retain native protein charges and generate the receptor structure file for molecular docking (Figure 1). The chemical structure of ECG was constructed using ChemDraw 2021 (Revvity Signals, Waltham, MA, USA), geometrically optimized with the MOPAC program [37], and exported as a PDBQT file for docking analysis (Figure 2b).

Structural energy optimization was performed using the Amber14 force field [38] in a two-step minimization strategy. Firstly, 1000 steps of structural optimization were conducted via the steepest descent method. Subsequently, further structural refinement was carried out with 500 steps of the conjugate gradient method. The final optimized structure was adopted as the model for subsequent molecular docking analysis.

ECG was used as the small-molecule ligand, and the myosin macromolecule was selected as the protein receptor. Molecular docking simulations were performed using the AutoDock 4.2.6 software suite (The Scripps Research Institute, San Diego, CA, USA). The configuration file was set to have a grid box with a size of 60 × 60 × 60 points, to cover the entire binding site, which was centered at the following coordinates, X: 86.187, Y: 37.926, and Z: 159.610. Docking iterations were set to 100, and all other parameters were set at default values. Generated binding poses were clustered based on the root-mean-square deviation (RMSD) of all ligand heavy atoms with a cluster cutoff of 5.00 Å, yielding 20 representative conformations. The pose with the lowest predicted binding free energy (kcal/mol) was selected according to the predefined scoring function in AutoDock 4.2.6.

### 2.13. Statistical Analysis

All graphs were plotted using Origin 9.0 software (Origin Lab Corporation, Northampton, MA, USA). Statistical significance was evaluated by one-way analysis of variance (ANOVA) followed by Duncan’s multiple range test (or Mann–Whitney U test) using SPSS Statistics 20.0 software (IBM Corporation, New York, NY, USA). Three independent extractions of myosin were performed from a single batch of beef, and each resulting myosin fraction was used to prepare corresponding ECG-myosin mixture samples for all treatment groups. Data are presented as mean ± standard deviation (*n* = 3, biological replicates). Differences were considered statistically significant at *p* < 0.05.

## 3. Results and Discussion

### 3.1. The Effect of ECG on the Antioxidant Properties of Myosin

#### 3.1.1. Oxidative Stability of the ECG–Myosin Mixtures

**Figure 3:**
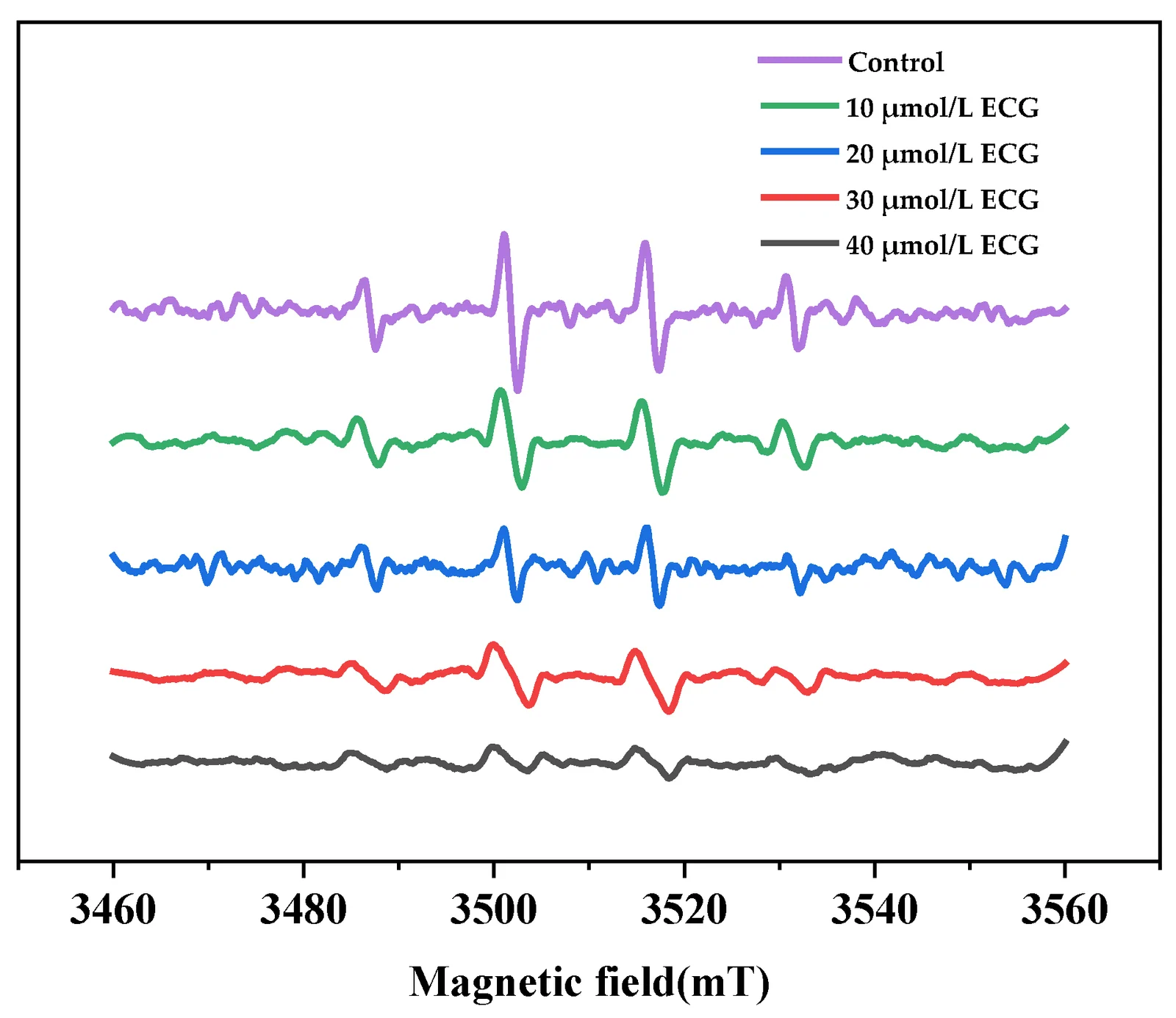
EPR-determined •OH signal intensities of samples across treatment groups.

The generation of hydroxyl radicals (•OH) was quantified via EPR spectroscopy to assess the antioxidant stability of ECG–myosin mixtures. The characteristic four-line (1:2:2:1) quartet EPR signal of DMPO–OH adduct was observed, which corresponds to the well-established spectral fingerprint of DMPO-trapped hydroxyl radicals. The signal intensity of hydroxyl radicals was represented by the overall amplitude of the EPR spectral envelope. As presented in Figure 3, pure myosin in the control group exhibited a distinct •OH signal under refrigerated storage at 4 °C, confirming that myosin is still susceptible to reactive oxygen species (ROS)-mediated oxidative damage even at low temperatures. In contrast, ECG–myosin mixtures exhibited markedly attenuated •OH signal intensities relative to the control, and this inhibitory effect was concentration-dependent. Increasing the ECG concentration from 10 to 40 μmol/L gradually diminished the EPR signals, and the •OH signal intensity was nearly undetectable at an ECG concentration of 40 μmol/L.

These results indicate that ECG binding improves myosin oxidative stability by effectively scavenging •OH, and alleviates ROS-induced protein oxidation. The present findings are consistent with those of Sharifimehr et al. [39], who observed significantly enhanced hydroxyl radical-scavenging capacity in tannic acid-conjugated faba bean protein, as reflected by the dose-dependent attenuation of EPR signal intensity. This further confirms the radical-quenching capability of polyphenol–protein interactions.

#### 3.1.2. Antioxidant Capacity of the ECG–Myosin Mixtures

**Figure 4:**
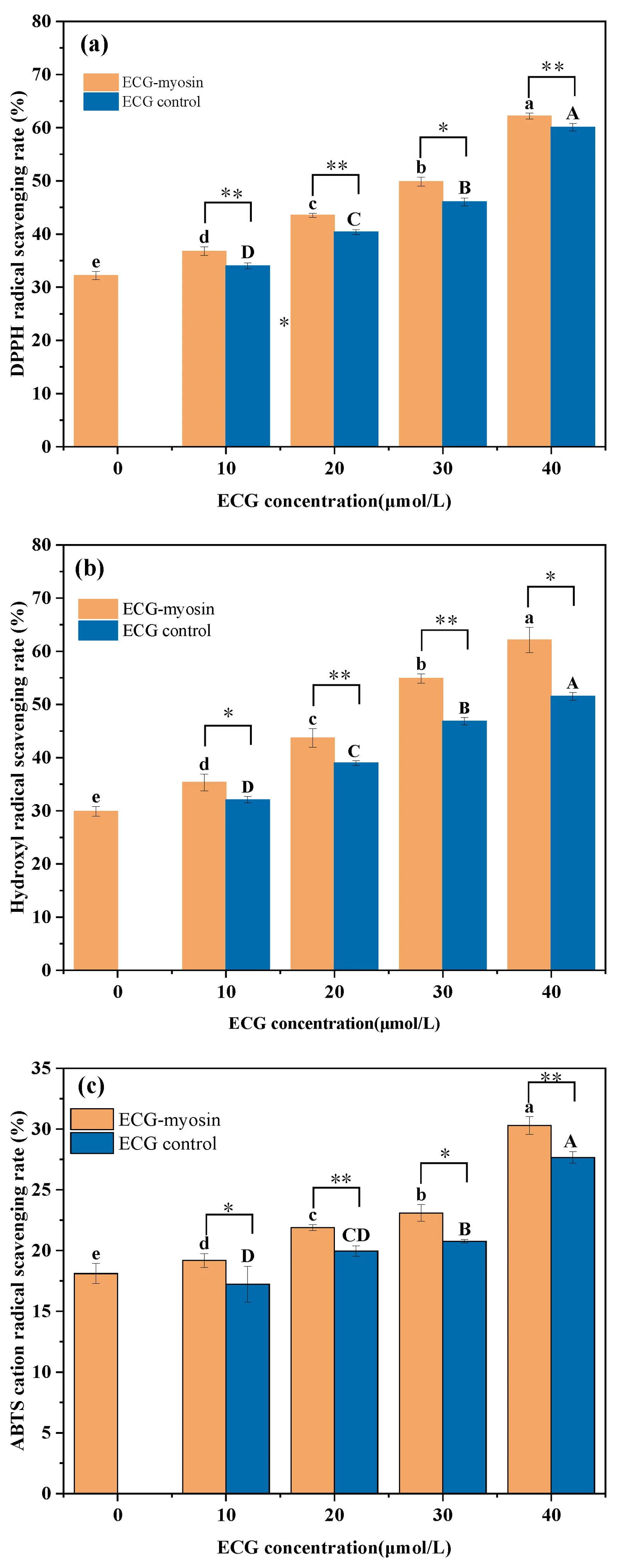
Effects of ECG–myosin mixtures with different ECG concentrations on free radical scavenging rate. (**a**) DPPH radical scavenging rate; (**b**) Hydroxyl radical scavenging rate. (**c**) ABTS cation radical scavenging rate. Duncan’s multiple range test was used to analyze differences among groups with different ECG concentrations at *p* < 0.05. Different lowercase letters (a–e) indicate significant differences between ECG concentration groups. Different uppercase letters (A–D) indicate significant differences among ECG control groups. The Mann–Whitney U test was applied to compare ECG control and ECG–myosin groups at the same concentration. * *p* < 0.05, ** *p* < 0.01.

The scavenging capacities of ECG–myosin mixtures against DPPH radicals, hy- droxyl radicals (•OH) were determined to evaluate the regulatory effects of ECG on the antioxidant capacity of myosin. As shown in Figure 4, the DPPH, hydroxyl radical scavenging and ABTS cation radical-scavenging rates of all ECG-myosin mixture groups were significantly higher than those of the pure myosin control (*p* < 0.05), and also markedly exceeded the values of ECG control groups at corresponding concentrations (*p* < 0.05). Relative to the ECG control groups with identical ECG concentrations, the ECG–myosin mixtures possessed stronger radical-scavenging capacities. Specifically, the maximum increment in DPPH radical scavenging rate was 8.33% (Figure 4a, *p* < 0.05). The highest increase in hydroxyl radical scavenging rate reached 20.54% (Figure 4b, *p* < 0.05), while the maximum enhancement of ABTS cation radical scavenging rate was 11.23% (Figure 4c, *p* < 0.05). These findings demonstrate that the binding of ECG to myosin confers a marked improvement in the antioxidant capacity of myosin. This observation is consistent with previous studies, in which complexes and conjugation of whey protein with polyphenol yielded enhanced antioxidant performance [40], and Pan et al. [41] reported superior DPPH radical scavenging capacity for covalent EGCG–bovine bone protein complexes. The above enhancement can be primarily attributed to phenolic hydroxyl groups originating from polyphenol moieties, which endow proteins with elevated hydrogen-donating potential, in addition to the establishment of intermolecular interactions between polyphenols and proteins [42,43].

### 3.2. The Effect of ECG on the Oxidation of the Amino Acid Side Chains of Myosin

#### 3.2.1. Sulfhydryl Group and Free Amino Group

**Figure 5:**
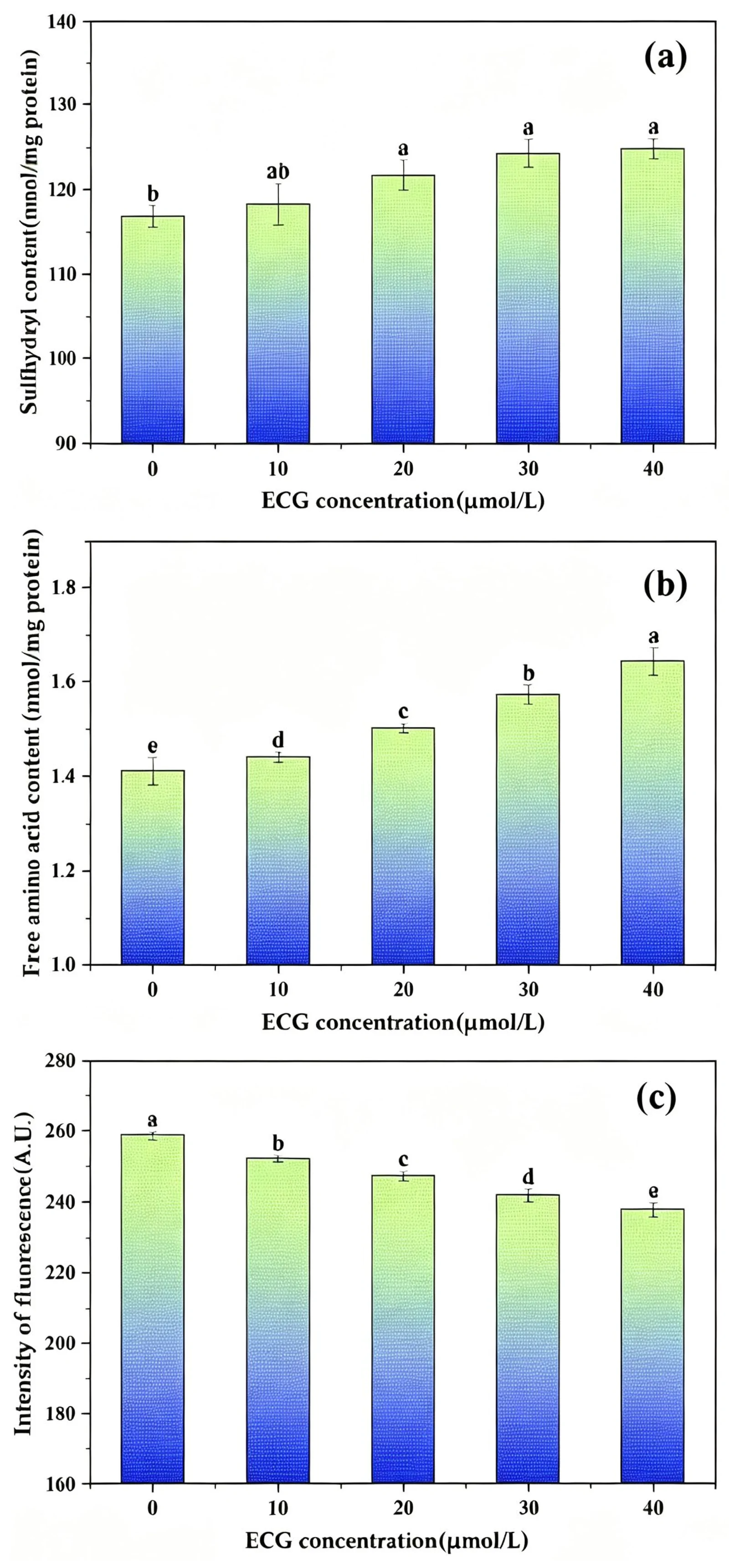
Effects of ECG–myosin mixtures with different ECG concentrations on amino acid side chain oxidation of bovine myosin. (**a**) Sulfhydryl content; (**b**) Free amino group content; (**c**) Dityrosine level.Different lowercase letters (a–e) indicate significant differences between ECG concentration groups (*p* < 0.05).

Amino acid side chains of myosin serve as critical targets for free radical-induced myosin oxidation. Accordingly, the effects of ECG binding on myosin oxidation and primary structure can be investigated by monitoring changes in amino acid side chains. The sulfhydryl content of freshly extracted myosin was determined to be 130.11 ± 2.08 nmol/mg protein. As shown in Figure 5a, following storage at 4 °C for 12 h, the sulfhydryl content of the control group decreased to 116.23 ± 1.16 nmol/mg protein, corresponding to 10.67% of sulfhydryl groups undergoing oxidative modification. Sulfhydryl contents in ECG–myosin mixtures were consistently higher than that of the control and increased with rising ECG concentration. The maximum sulfhydryl content (124.95 ± 1.15 nmol/mg protein) was observed in the 40 μmol/L ECG treatment group, which was significantly higher than the control (*p* < 0.05).

The trend of free amino group content across all treatment groups resembled that of sulfhydryl groups, as shown in Figure 5b. Free amino group levels gradually increased with elevated ECG concentration and were significantly higher than those of the control (*p* < 0.05). These results demonstrate that ECG effectively alleviated oxidative modification of free amino groups in myosin.

#### 3.2.2. Dityrosine

Dityrosine is generated upon oxidation of tyrosine residues from two separate or identical polypeptide chains, which triggers intra- and intermolecular protein cross-linking [44]. Dityrosine levels are positively correlated with the degree of protein oxidation. As shown in Figure 5c, the dityrosine levels of myosin in the control group reached 258.56 A.U. Following complexation with ECG, dityrosine levels were significantly reduced in a dose-dependent manner; the minimum value (231.83 A.U.) was observed for the 40 μmol/L ECG-treated group. These findings confirmed that ECG efficiently suppressed tyrosine oxidation.

Collectively, these findings indicate that the binding of ECG to myosin efficiently suppresses sulfhydryl oxidation, free amino group oxidation, and dityrosine generation in a concentration-dependent manner. This protective effect can be attributed to the polyhydroxyl hydrogen-donating groups in ECG, which neutralize reactive oxygen species and other free radicals, thereby mitigating myosin oxidation. The ability of polyphenols to preserve protein sulfhydryl and free amino groups has been extensively reported in previous research. For instance, mulberry leaf extracts rich in polyphenols and gallic acid effectively retarded sulfhydryl depletion in fresh meat myofibrils [45,46]. Likewise, quercetin and caffeic acid prevented the oxidation of sulfhydryl groups into disulfide bonds in sausage myosin [47].

### 3.3. Effects of ECG on Myosin Secondary Structure

To further elucidate the effects of ECG on the conformational structure of beef myosin and the underlying interaction mechanism, changes in the secondary and tertiary structures of myosin induced by ECG were systematically investigated.

#### 3.3.1. Circular Dichroism

**Figure 6:**
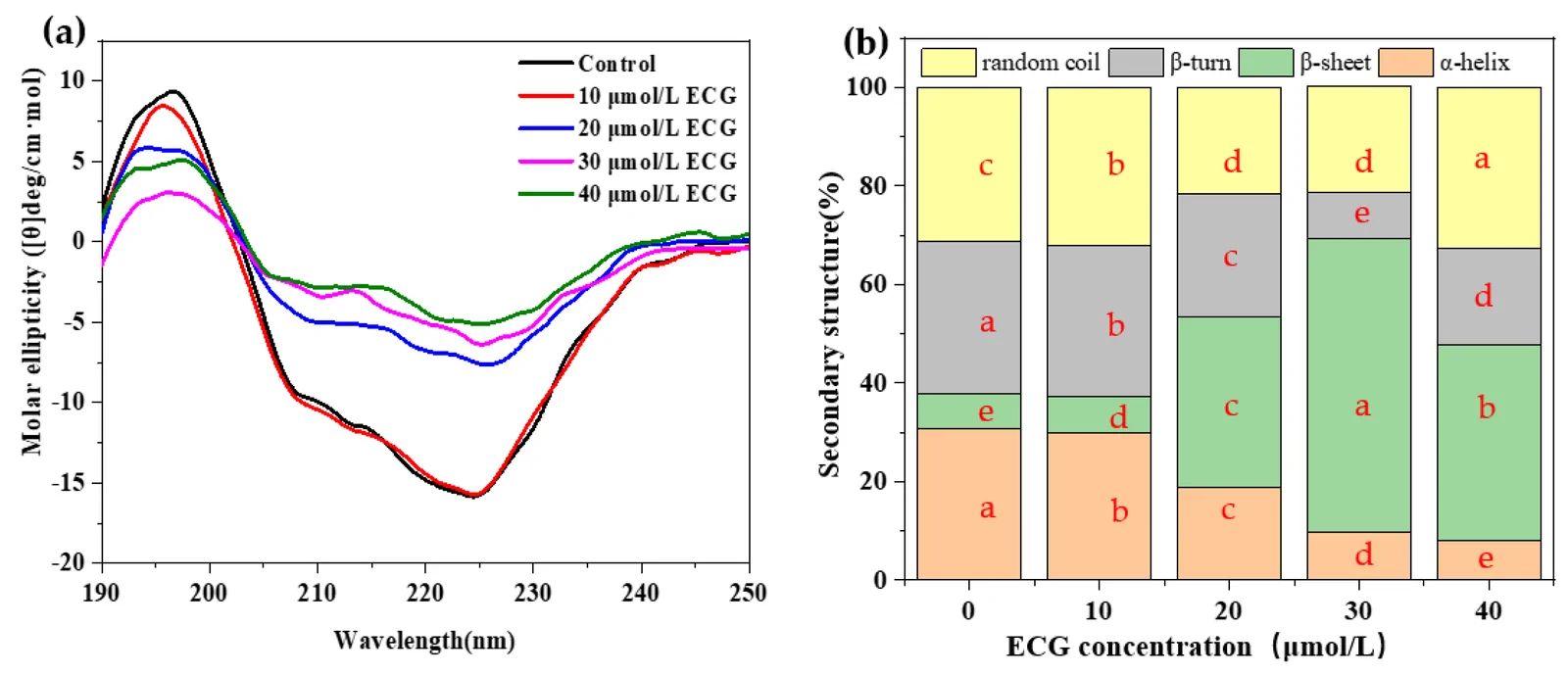
The effect of ECG on the secondary structure of myosin. (**a**) Far-UV circular dichrogram spectra; (**b**) content change of secondary structure.Different lowercase letters (a–e) indicate significant differences between ECG concentration groups (*p* < 0.05).

The secondary structure composition of ECG–myosin mixtures was analyzed by circular dichroism spectroscopy and the NRMSD values for all fitted spectra were below 0.15, indicating reliable fitting performance. As illustrated in Figure 6a, the spectrum of the control group displayed a prominent characteristic absorption peak at 228 nm, which corresponded to the typical spectral feature of myosin α-helical structure. Following binding with 10 μmol/L ECG, no obvious shift or attenuation was detected in the CD curve. In contrast, raising the ECG concentration to 20 μmol/L markedly reduced the intensity of the 228 nm characteristic peak. Further increases in ECG concentration resulted in a gradual reduction in peak intensity, suggesting a progressive loss of the α-helix content in myosin.

Quantitative variations in the secondary structure fractions, as shown in Figure 6b, further validated this trend: the α-helix proportion declined with rising ECG concentration. As the ECG concentration increased from 10 to 30 μmol/L, β-sheet content increased markedly (*p* < 0.05) and was significantly higher than those of the control (*p* < 0.05). Notably, compared with the 30 μmol/L ECG-treated group, the 40 μmol/L ECG treatment induced a remarkable drop in β-sheet content, accompanied by simultaneous elevation in the proportions of β-turn and random coil structures. This structural transition pattern may be attributed to the intrinsic binding affinity and interaction characteristics between ECG and myosin.

Collectively, ECG exerts a biphasic effect on the secondary structure of myosin. At ECG concentrations of 10–30 μmol/L, ECG induces the transition from α-helix to β-sheet in bovine myosin and promotes conformational rearrangement of myosin in a dose-dependent manner. However, treatment with 40 μmol/L ECG reduces the stability of myosin secondary structure, which may be related to the nature and strength of the interaction between ECG and myosin. These findings highlight the necessity of selecting an optimal ECG dosage for the antioxidant preservation of meat and meat products.

#### 3.3.2. FTIR

**Figure 7:**
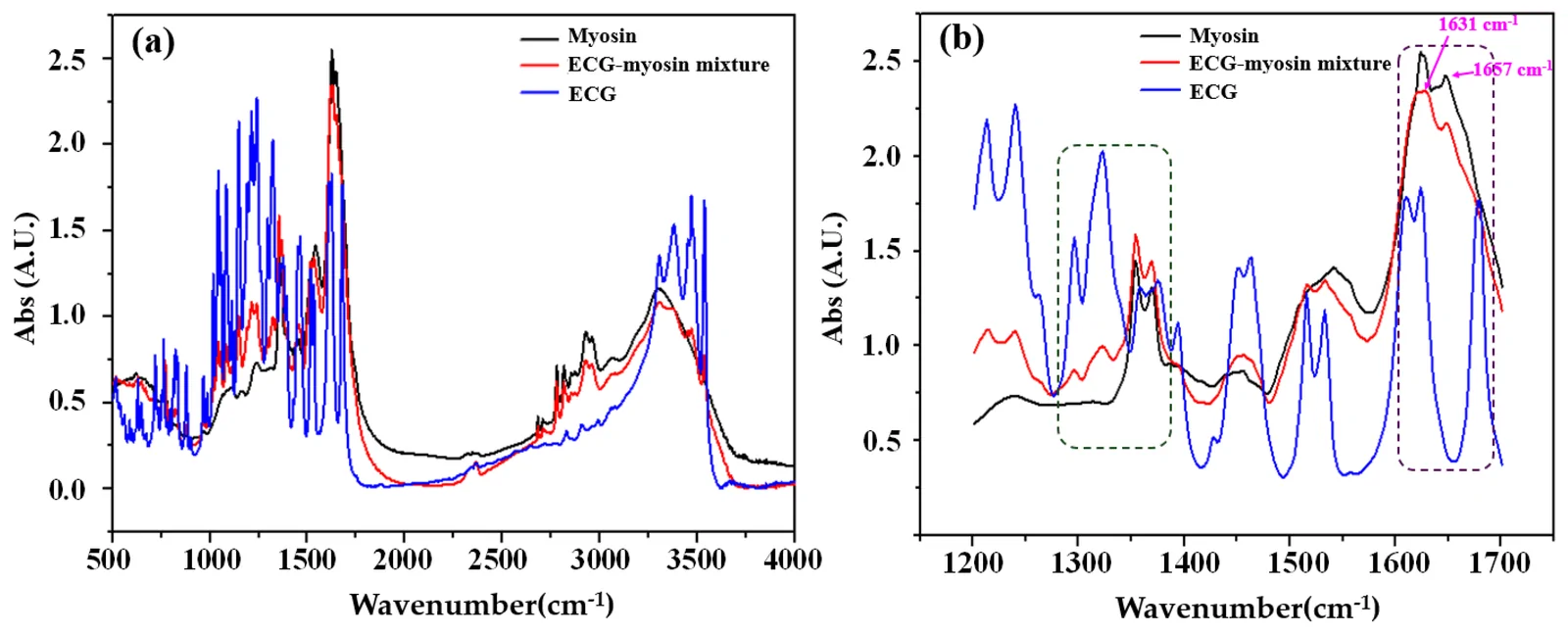
FTIR spectra of ECG, myosin and their mixture (5:5). (**a**) FTIR spectra (4000–500 cm^−1^); (**b**) FT IR spectra (1700–1200 cm^−1^).

FTIR spectroscopy is a standard technique for characterizing protein secondary structures, which are categorized into four main conformations: β-sheet (1640–1610 cm^−1^), random coil (1650–1640 cm^−1^), α-helix (1658–1650 cm^−1^), and β-turn (1700–1660 cm^−1^) [48]. In the present study, FTIR spectroscopy was applied to further identify binding sites between ECG and myosin and characterize corresponding changes in myosin secondary structure. FTIR spectra of ECG, pure myosin, and the 30 μmol/L ECG–myosin mixture (5:5, *w*/*w*) are displayed in Figure 7. No new absorption peaks were detected in the spectra of the ECG–myosin mixture, which indicates that the ECG–myosin interaction may occur mainly via non-covalent forces. The ECG–myosin mixture exhibited drastically reduced absorbance intensity within the 3584–3283 cm^−1^ region, which corresponds to O–H stretching vibrations of ECG. This attenuation indicates that the O–H stretching vibration of ECG was weakened following non-covalent binding, implying that ECG acts as a hydrogen bond donor and facilitates hydrogen bond formation within the ECG–myosin mixture. Subsequent analyses focused on spectral variations within the amide I band of protein samples.

The amide I band spectra of ECG, myosin, and the ECG–myosin mixture (5:5, *w*/*w*) are presented in Figure 7b. The overall spectral profile of the ECG–myosin mixture closely matched that of pure myosin, indicating the polypeptide backbone retained its native conformation. Notably, the characteristic absorption band of ECG at 1675 cm^−1^ [49] underwent a prominent red shift to 1640–1620 cm^−1^ after complex formation, along with increased peak absorbance intensity. Meanwhile, the absorption peak at 1657 cm^−1^ for the ECG–myosin mixture decreased compared with that of the pure myosin. This spectral shift indicates that the content of β-sheet increased while the α-helix content experienced reduction [50], which is consistent with the results shown in Figure 6b from CD analysis.

**Figure 8:**
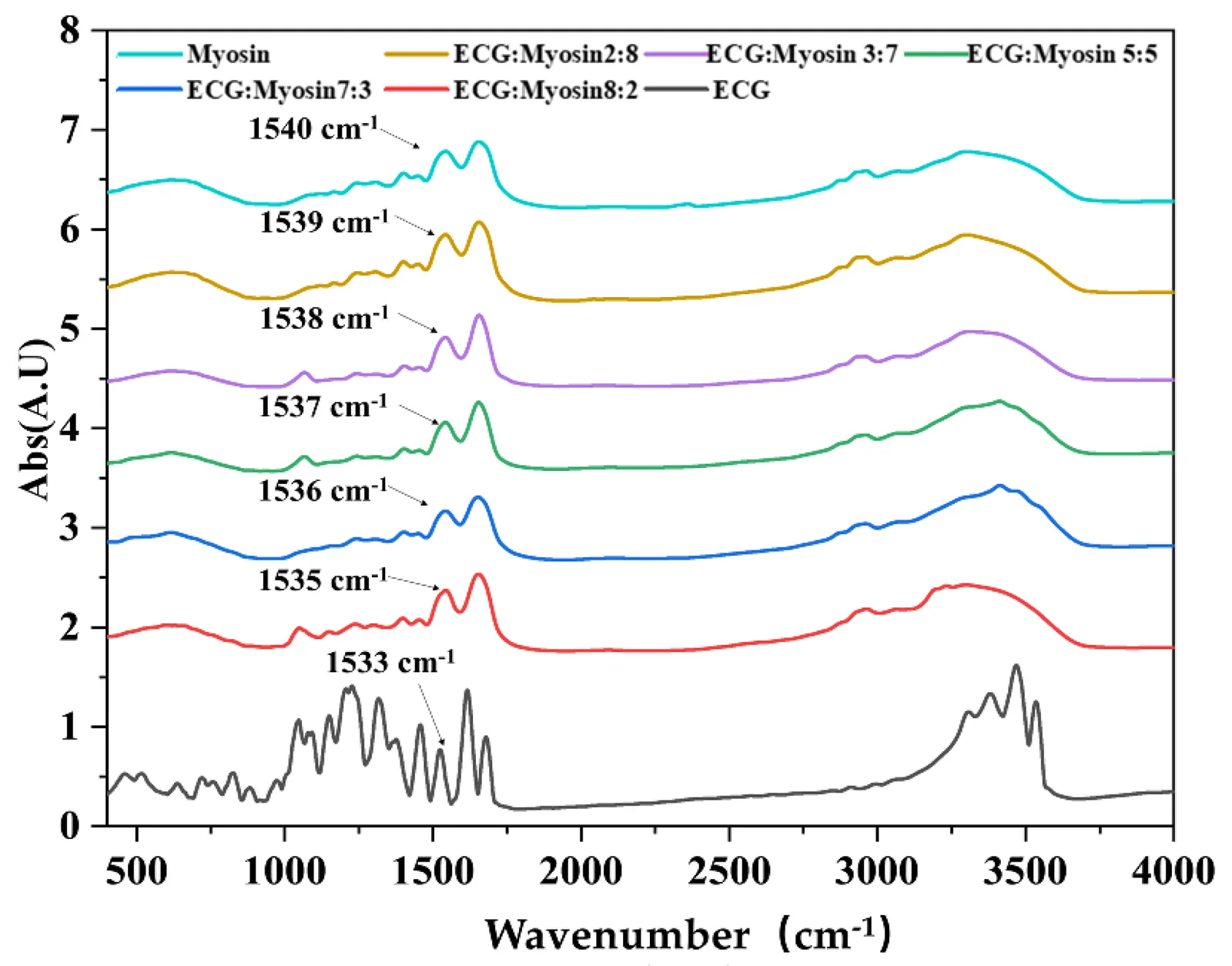
FTIR spectra of ECG, myosin and their mixture of different proportions.

As shown in Figure 8, in the amide II region (1600–1500 cm^−1^), myosin exhibited a characteristic absorption band of pure myosin at 1540 cm^−1^, originating from C–N stretching and N–H bending vibrations, whereas ECG exhibited a distinct peak at 1533 cm^−1^. The corresponding band of the ECG–myosin mixture (5:5) shifted to 1537 cm^−1^; this peak was blue-shifted (peak shifts to higher wavenumber) compared with pure myosin, yet red-shifted (peak shifts to lower wavenumber) relative to isolated ECG. This spectral shift demonstrates that ECG–myosin interactions remodeled the local microenvironment and gradually displaced the amide II absorption band toward lower wavenumbers.

The observed spectral perturbations arise from two primary factors. On the one hand, the phenolic hydroxyl moieties of ECG act as potent hydrogen bond donors and may form intermolecular hydrogen bonds with the carbonyl (C=O) groups of myosin. This interaction elevates the total hydrogen-bonding density and lowers the C=O bond order, which triggers a red shift of the corresponding vibrational frequency [50]. On the other hand, hydrophobic interactions between the aromatic scaffolds of ECG and nonpolar domains of myosin generate vibrational coupling of ECG’s C=C skeletal stretching signals, as evidenced by broadening and displacement of absorption bands across the 1620–1517 cm^−1^ region. These spectral variations verify that the aromatic ring framework of ECG contributes to the binding interface.

The amide III band (1330–1220 cm^−1^) is free from water absorption and O–H interference, enabling unambiguous separation of α-helix and random coil signals, thus providing valuable supplementary information on protein conformational rearrangements. As displayed in Figure 7b, the absorption peak at 1322 cm^−1^ within the amide III region corresponds to the O–H deformation vibration of phenolic hydroxyl groups in ECG; this peak exhibits strong absorbance intensity in pure ECG. For the ECG–myosin mixture, the intensity of this peak was markedly weakened, which is most likely caused by the breakage of O–H bonds upon interaction between ECG and myosin. Alternatively, the observed reduction in peak intensity may originate from vibrational coupling between the hydroxyl moieties of ECG and the –CH_2_ bending vibration of myosin, which reduces the apparent signal intensity originating from O–H groups.

To sum up, the characteristic shifts in amide I and amide II bands indicate that ECG binds to myosin mainly through non-covalent forces dominated by hydrogen bonding and hydrophobic association, accompanied by subtle yet functionally essential conformational rearrangements in the secondary structure of myosin. Furthermore, the absorption changes in the amide III region reveal that the phenolic hydroxyl groups of ECG participate in hydrogen bond formation during its binding with myosin. The above results will be further verified in the subsequent analysis.

### 3.4. Intrinsic Fluorescence Spectroscopy

**Figure 9:**
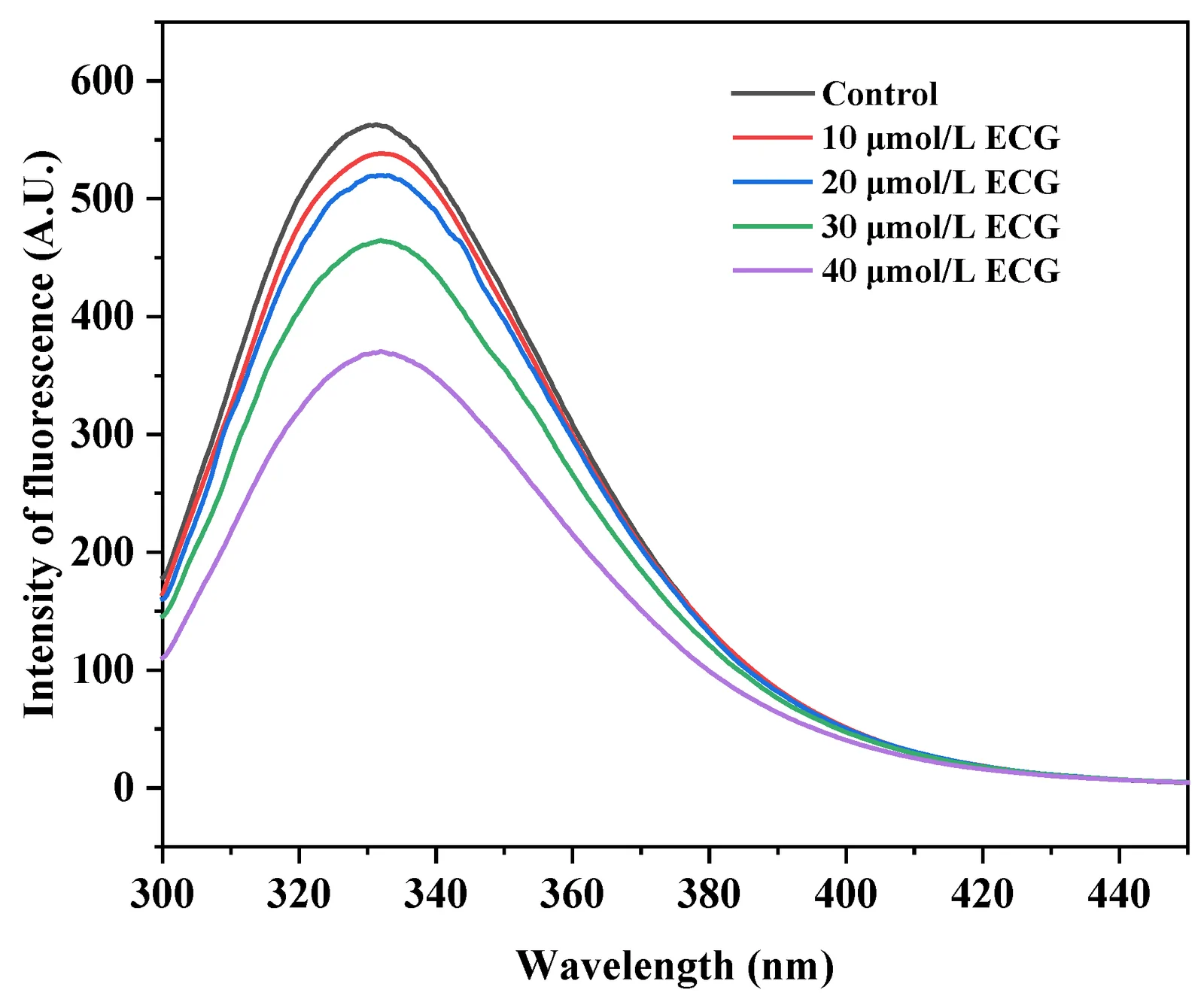
Effect of ECG on the fluorescence intensity of myosin.

Endogenous fluorescence mainly originates from tryptophan (Trp) and tyrosine (Tyr) residues of myosin, and its intensity is highly sensitive to the microenvironment surrounding these aromatic residues, which can reflect conformational alterations in the protein tertiary structure. As shown in Figure 9, the fluorescence quenching rates of 10, 20, 30, and 40 μmol/L ECG were 3.74%, 6.81%, 16.71%, and 33.44%, respectively, compared with the control. These results confirm concentration-dependent quenching of myosin by ECG, demonstrating direct molecular interactions. ECG binds to Trp and Tyr residues of myosin via hydrophobic interactions and disrupts the hydrophobic microenvironment of fluorophores, triggering fluorescence quenching. This interaction pattern is consistent with previous reports describing the binding behavior between catechin derivatives and meat-derived myosin [51]. Meanwhile, formation of non-fluorescent ECG-myosin ground-state complexes may also account for fluorescence quenching. At 40 μmol/L ECG, quenching was greatly intensified (33.44%). High-concentration ECG contains more phenolic hydroxyl groups, conferring stronger hydrophobic interactions and hydrogen-bond-forming capacity, which enhances its fluorescence-quenching ability and leads to stronger interaction between ECG and myosin. Meanwhile, excessive ECG induces partial unfolding of the tertiary structure of myosin; partially exposed buried Trp residues change the microenvironment of fluorophores and further aggravate quenching.

Previous studies have reported distinct red-shifts in the maximum emission wavelength upon polyphenol treatment, which is interpreted as exposure of aromatic residues to polar solvent. Whereas only a slight red shift in the peak wavelength of the ECG–myosin mixtures was observed in the present study. This discrepancy can be ascribed to variations in protein sources, buffer conditions, and applied ECG concentration ranges [52].

### 3.5. Surface Hydrophobicity

**Figure 10:**
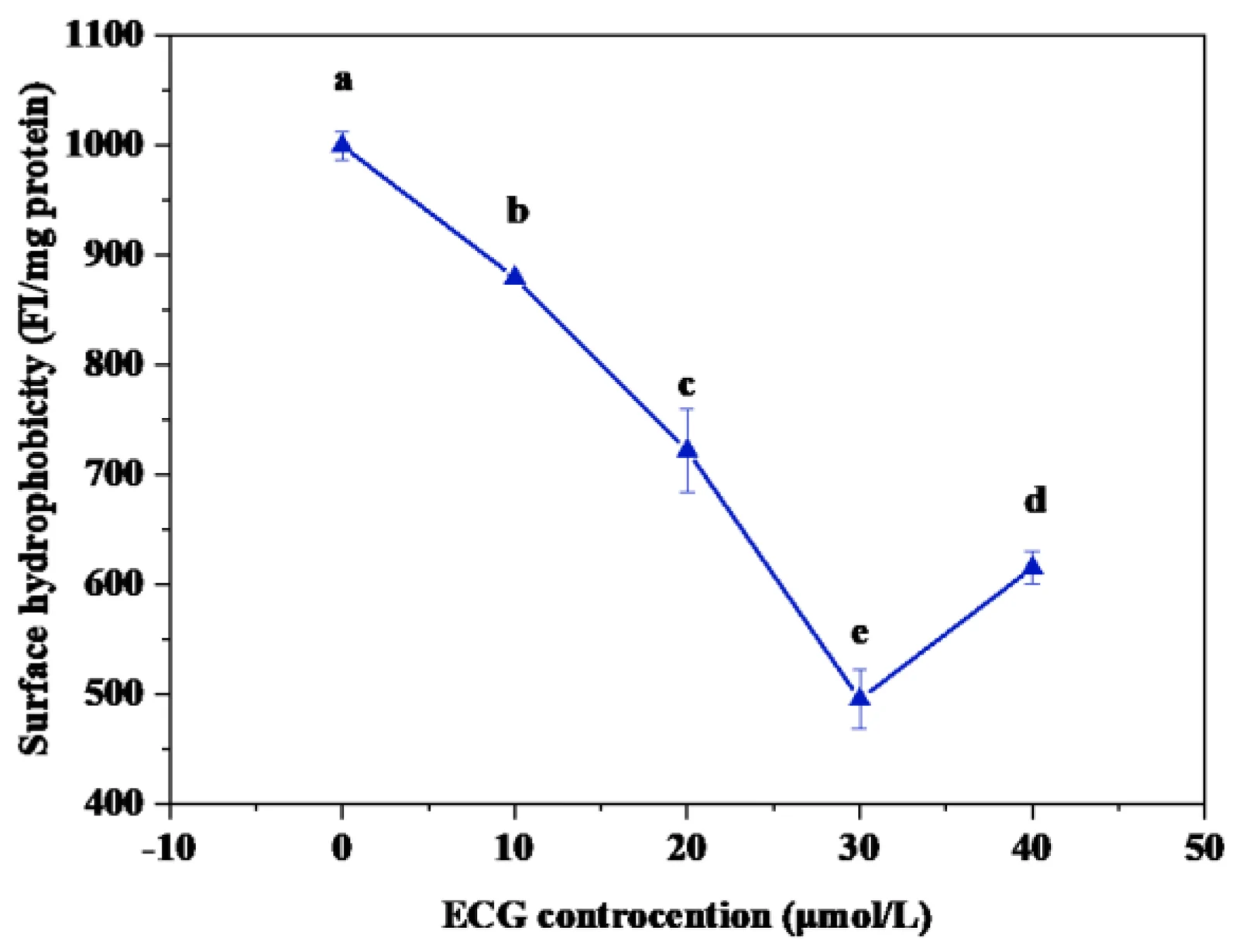
Effects of ECG on the surface hydrophobicity of myosin.Different lowercase letters (a–e) indicate significant differences between ECG concentration groups (*p* < 0.05).

Protein surface hydrophobicity is closely associated with the exposure extent of hydrophobic amino acid residues, such as Trp. The exposure of hydrophobic moieties can be induced by protein oxidation or polyphenol-mediated conformational alterations of proteins. A higher degree of surface hydrophobicity indicates a greater extent of unfolding of the tertiary structure of proteins [53]. As depicted in Figure 10, the surface hydrophobicity of myosin in the control group was determined to be 999.15 FI/mg protein. With increasing ECG concentrations from 0 to 30 μmol/L, the surface hydrophobicity of myosin exhibited a significant downward trend (*p* < 0.05). Specifically, the surface hydrophobicity of the ECG–myosin mixture decreased to 495.08 FI/mg protein at an ECG concentration of 30 μmol/L, corresponding to a 50.45% reduction relative to the control group.

One plausible explanation is that ECG retarded myosin oxidation. As shown in Section 3.2.1, compared with freshly isolated myosin, myosin stored at 4 °C for 12 h displayed lower sulfhydryl content and a thicker 250 kDa band in SDS-PAGE (Figure S1), indicating that myosin is prone to oxidative modification shortly after extraction. Surface-accessible hydrophobic clusters reside in the myosin head domain. This domain is rich in sulfhydryl groups and more susceptible to oxidation than the tail region [41]. Oxidation of the myosin head triggers structural unfolding and exposes buried hydrophobic residues. By contrast, ECG (10, 20 and 30 μmol/L) inhibited oxidation-induced hydrophobic exposure and decreased surface hydrophobicity.

This decline also arises from direct ECG-myosin binding. Benefiting from their aromatic benzene-ring skeleton, polyphenols bind to exposed hydrophobic amino acid residues of myosin (e.g., Trp, Tyr, leucin) and occupy surface hydrophobic sites, decreasing the number of accessible hydrophobic sites [50]. Additionally, polyphenols possess abundant hydroxyl groups; upon protein binding, these groups improve the hydrophilic environment of the protein and further reduce surface hydrophobicity [22]. Similar findings have been documented for meat proteins treated with phenolic compounds [54].

Notably, when the ECG concentration further increased to 40 μmol/L, the surface hydrophobicity of the ECG–myosin mixture recovered to 615.09 FI/mg protein. This may be attributed to the fact that, at this high concentration, excess ECG molecules could act as intermolecular linkers to induce mild cross-linking among myosin molecules, resulting in the aggregation of hydrophobic groups on the protein surface. Alternatively, high-concentration ECG (40 μmol/L) could markedly enhance the quenching intensity of myosin (Figure 8), suggesting that excessive ECG binding might aggravate the perturbation of myosin tertiary structure. This can trigger partial unfolding or conformational changes, exposing a large number of originally buried hydrophobic residues to the aqueous phase and thereby significantly increasing surface hydrophobicity. This observation is consistent with the structural variation trends described in Section 3.3.1, in which a higher proportion of disordered structures (random coils and β-turns) was detected in myosin treated with 40 μmol/L ECG. In line with the present results, Jia et al. [55] found that low-dose catechins (10 μmol/g) reduced turbidity and improved the solubility of pork myosin, whereas high-dose catechins (50 μmol/g) significantly increased its surface hydrophobicity.

### 3.6. Effects of ECG on the Microstructure of Myosin

**Figure 11:**
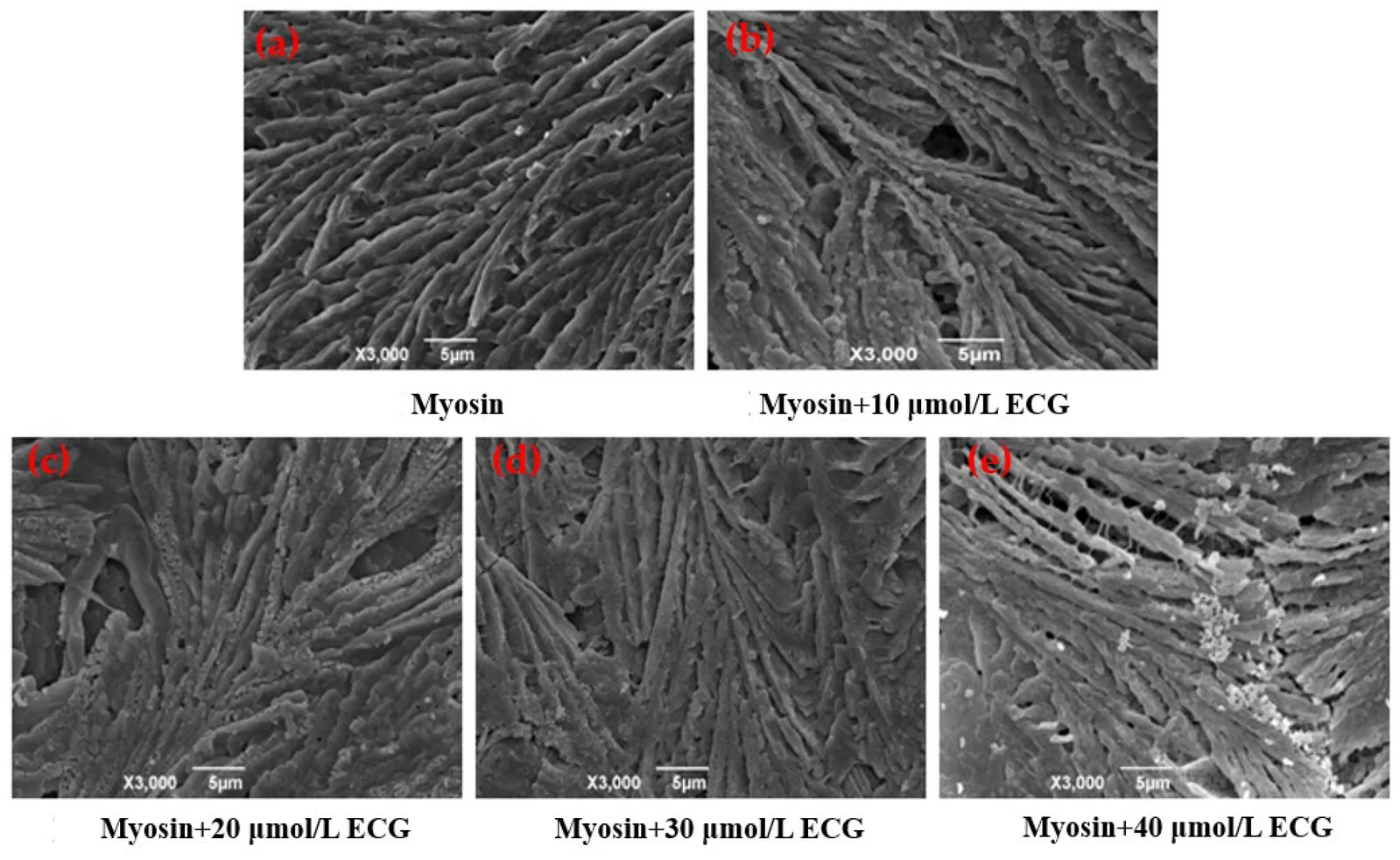
Effects of ECG on micromorphology of myosin (×3000).

As presented in Figure 11, the myofibril bundles assembled from myosin exhibited uniform and well-ordered arrangement in the control group (Figure 11a). No obvious alterations in the organization of myofibril bundles of ECG–myosin mixtures were observed upon treatment with ECG at concentrations of 10 and 20 μmol/L (Figure 11b,c). Nevertheless, the myofibril bundles became less ordered in the 30 μmol/L ECG-treated group (Figure 11d). When the ECG concentration was further increased to 40 μmol/L, the myofibril bundles of ECG–myosin mixtures displayed disorganized and chaotic arrangement, accompanied by pronounced aggregation and fragmentation (Figure 11e). These results indicate that high-concentration ECG (40 μmol/L) can trigger cross-linking and aggregation of myosin, thereby inducing structural disorder. Consistent with the present findings, previous studies have reported that low-dose tea polyphenols (TP) induce mild aggregation of beef myofibrillar proteins (MPs), whereas high-dose TP causes excessive aggregation and impairs the functional properties of beef MPs [41]. Besides, excess ECG and EGCG led to cross-linked aggregation and prevented the formation of gel networks [22]. The above observations further highlight that careful selection of appropriate concentration ratios is critical when polyphenols are employed to ameliorate the oxidative-stability and functional properties of proteins.

### 3.7. ITC

**Figure 12:**
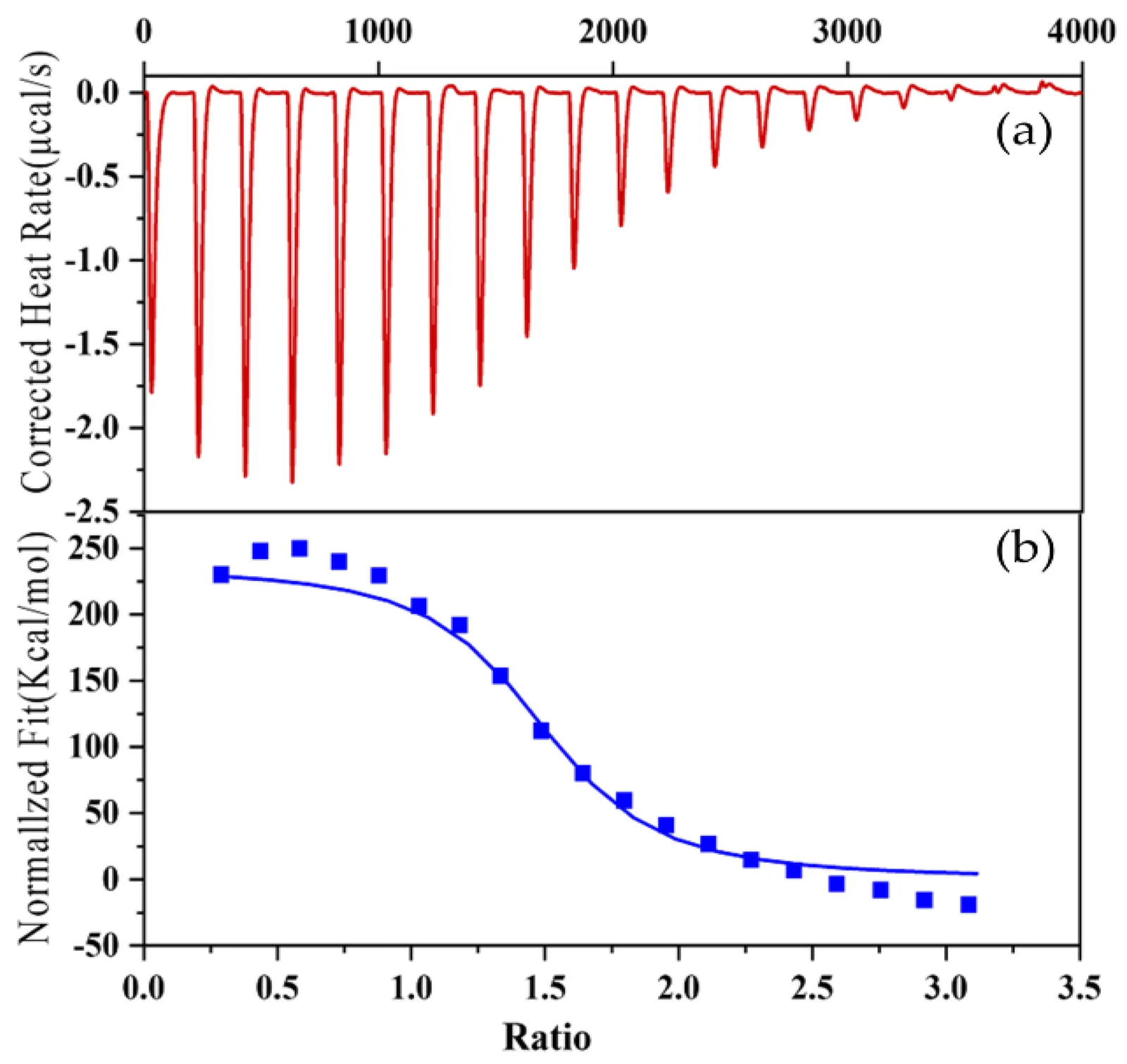
ITC caloric change curve of interaction between 400 μmol/L ECG and 20 μmol/L myosin. (**a**) raw heat-time curve; (**b**) heat curve.

The non-covalent binding mechanism between ECG and myosin was investigated via ITC. At 4 °C, 400 μmol/L ECG was titrated into 20 μmol/L myosin solution, and the raw heat–time curve was presented in Figure 12a. According to the peak profile, the downward peak demonstrates that the non-covalent binding between ECG and myosin was endothermic (Δ*H* > 0). With the continuous titration of ECG into myosin solution, the reaction heat gradually decreased and approached zero when the binding reaction reached saturation. Subsequently, raw ITC data were analyzed with Nano Analyze 2021 software. After correcting for the solvent blank and dilution heat produced during titration, the heat curve was fitted to a one-site binding model, generating a typical sigmoidal (“S-shaped”) binding curve (Figure 12b). Thermodynamic binding behavior between ECG and myosin was quantitatively evaluated using the calibrated ITC titration curves. The valid datasets exhibited good fits to the single-site binding model. Low and stable RSS values for fitted curves confirmed the reliable performance of this nonlinear binding-model fitting (Table S4). The corresponding thermodynamic parameters for ECG–myosin binding at 4 °C were determined as follows: binding constant *Ka* = (1.52 ± 0.17) × 10^7^ L/mol, binding stoichiometry *n* = 1.311 ± 0.115, Δ*H* = 95.99 ± 5.21 kJ/mol, Δ*S* = 483.83 ± 17.28 J/(mol·K), and Δ*G* = −38.08 ± 0.34 kJ/mol. The relatively high *Ka* value indicates a strong binding affinity between ECG and myosin. The stoichiometry close to 1.0 further supports the suitability of the single-site binding model for this interaction system, although the slightly higher *n* value may imply minor non-specific binding. These results verified that the binding of ECG to myosin at 4 °C was entropy driven (Δ*S* > 0), endothermic (Δ*H* > 0), and thermodynamically spontaneous (Δ*G* < 0). The positive ΔH and ΔS values collectively indicate that hydrophobic interactions serve as the predominant non-covalent driving force for ECG–myosin binding. As ECG titration proceeded, hydrophobic interactions between ECG and myosin were progressively formed. The gradual decay of reaction heat toward zero signified that the binding reaction approached saturation, implying that hydrophobic binding sites on myosin became fully occupied by ECG molecules at the end of titration. Consistent with our findings, Frazie et al. also reported that hydrophobic forces dominated the non-covalent interactions between catechins and casein based on ITC measurements [56].

### 3.8. Force of Interaction Analysis

**Figure 13:**
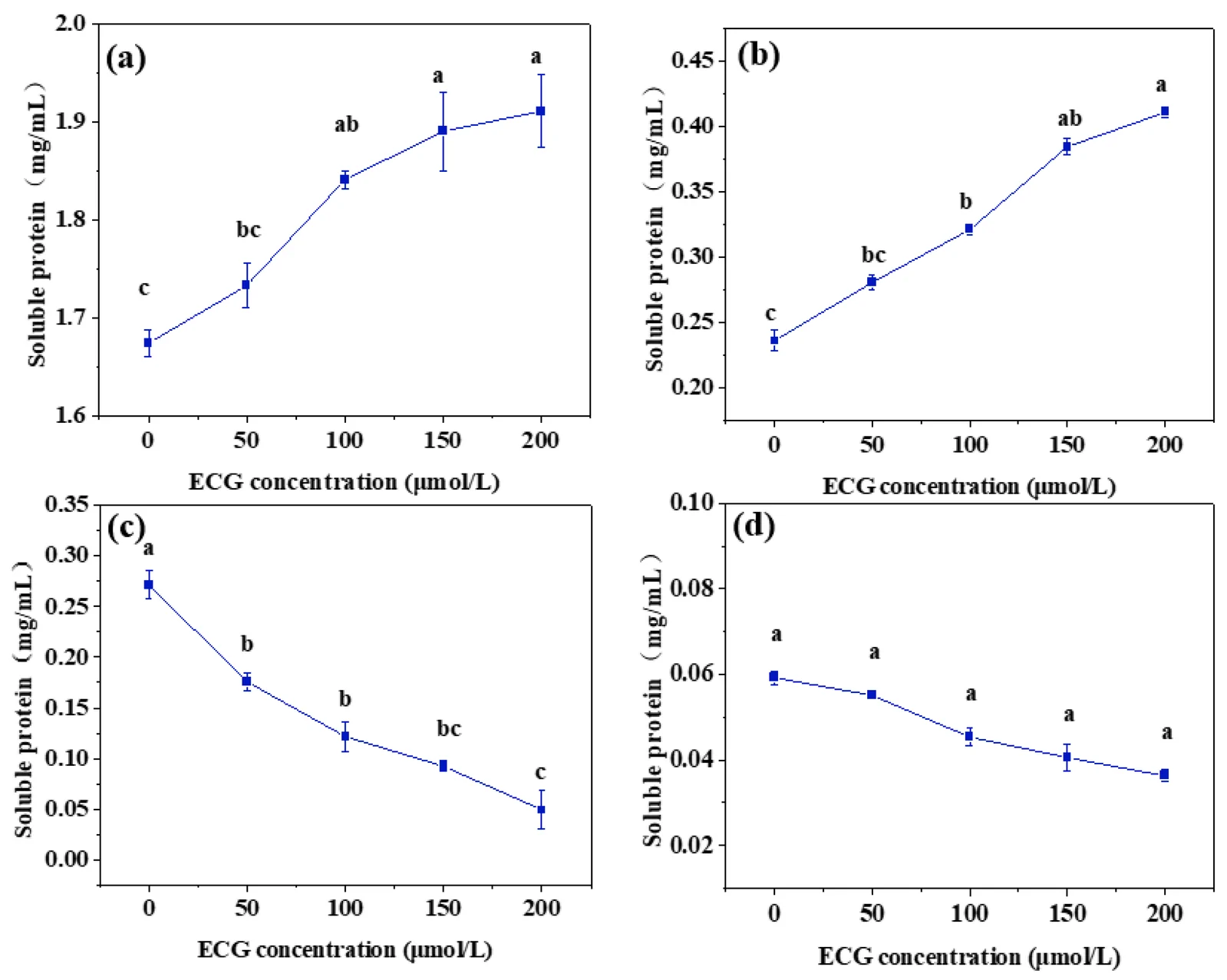
Changes in chemical forces between ECG and myosin. (**a**) hydrophobic interaction; (**b**) hydrogen bonding; (**c**) ionic bonds; (**d**) disulfide bonds.Different lowercase letters (a–c) indicate significant differences between ECG concentration groups (*p* < 0.05).

Non-covalent protein–polyphenol interactions are generally driven by multiple synergistic forces [49]. Therefore, an additive perturbation assay was performed to further identify the major non-covalent interactions between ECG and myosin. The changes in chemical forces for each sample group are shown in Figure 13. With increasing ECG concentration, hydrophobic interactions and hydrogen bonding were gradually enhanced, ionic bonding was weakened, while disulfide bond contents exhibited no significant changes. When the ECG concentration reached 200 μmol/L, hydrophobic interaction and hydrogen bonding were significantly increased by 14.13% (*p* < 0.05) and 74.06% (*p* < 0.05), respectively, compared with myosin samples without ECG addition in the control group, whereas ionic bonds decreased significantly by 81.86%. There was still no significant change in disulfide bond contents. These results indicate that ECG addition markedly strengthened hydrophobic and hydrogen-bond interactions while weakening ionic-bond interactions. Hence, hydrophobic and hydrogen-bond interactions serve as the driving forces for non-covalent ECG–myosin binding. This finding agrees with earlier reports that hydrophobic interactions dominate protein–polyphenol complex formation under refrigerated storage conditions [17,50].

### 3.9. Molecular Docking

**Figure 14:**
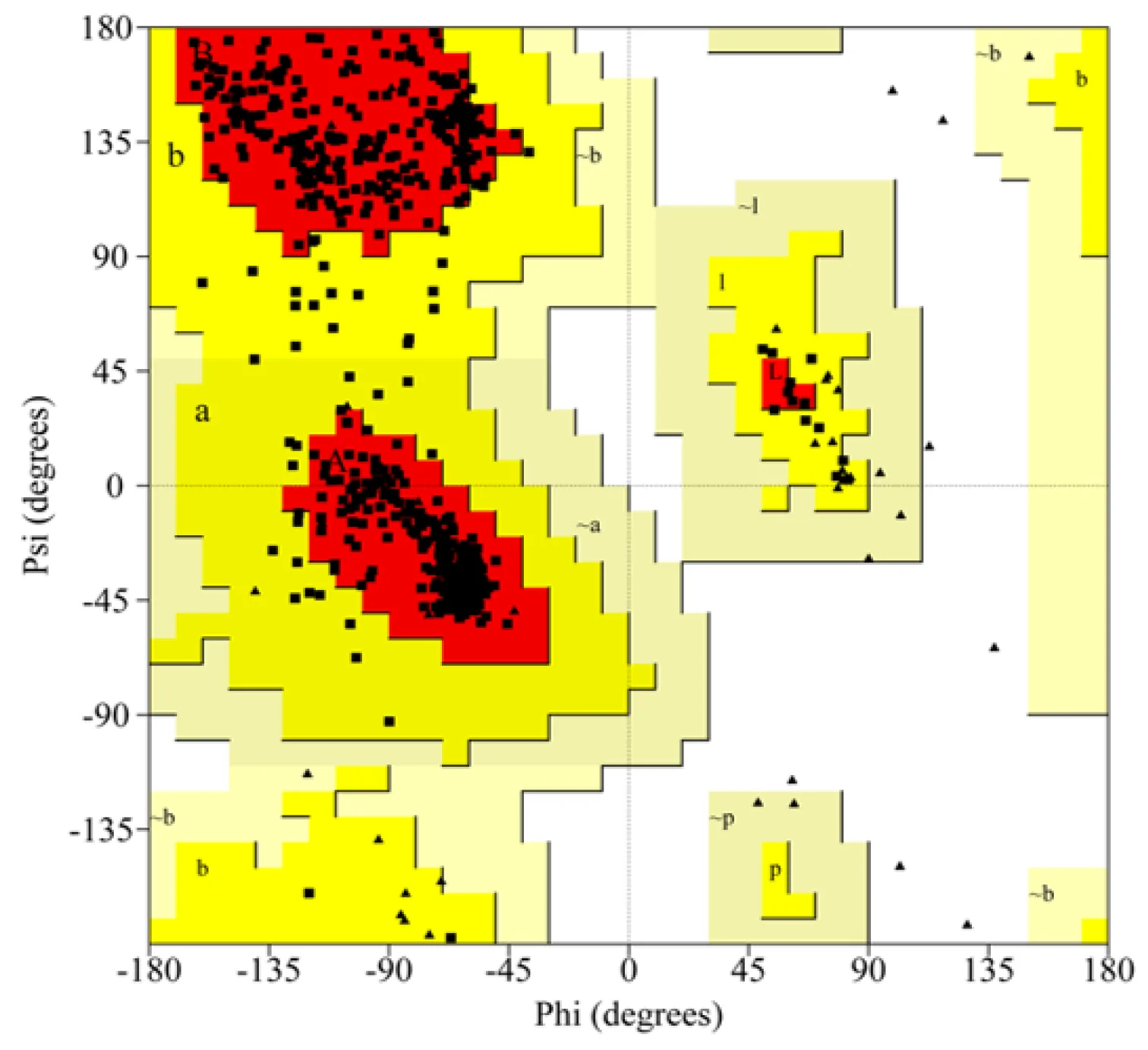
The Ramachandran plot for the homology model. (Red region: most favored regions; yellow region: additional allowed regions; A: most favored regions for α-helix; B: most favored regions for β-sheet; a: additional allowed region for α-helix; b: additional allowed region for β-sheet; p: allowed region for Polyproline II (PPII) conformation; ~a, ~b, ~p, ~I:generously allowed region).

Molecular docking represents a powerful technique for visualizing the binding behavior and intermolecular interactions between small-molecule ligands and macromolecular proteins [57], which is used to further explore how myosin interacts with ECG to confirm interaction sites. The completed and optimized protein model was evaluated through the procedure “PROCHECK” [58]. When the percentage is >90%, the model is valid. Figure 14 shows online assessment of the myosin homology modeling model. In this study, 92.70% of the amino acid residues are located in most favored regions (red areas in Figure 14), and 7.10% of the amino acid residues are located in additional allowed regions (yellow areas in Figure 14). The total percentage of amino acids residues in the reasonable range was 99.80%, which indicates that all dihedral angles of the amino acid residues in the modeled myosin protein fall within reasonable ranges, consistent with stereochemical energy rules. Therefore, the model was considered reliable and was further used for molecular docking studies. Blind docking was selected due to the unknown protein binding site, and the docking position with the lowest binding energy was chosen as the most representative site. The best pose with the lowest binding energy (−9.864 kcal/mol) is shown in Figure 1.

**Figure 15:**
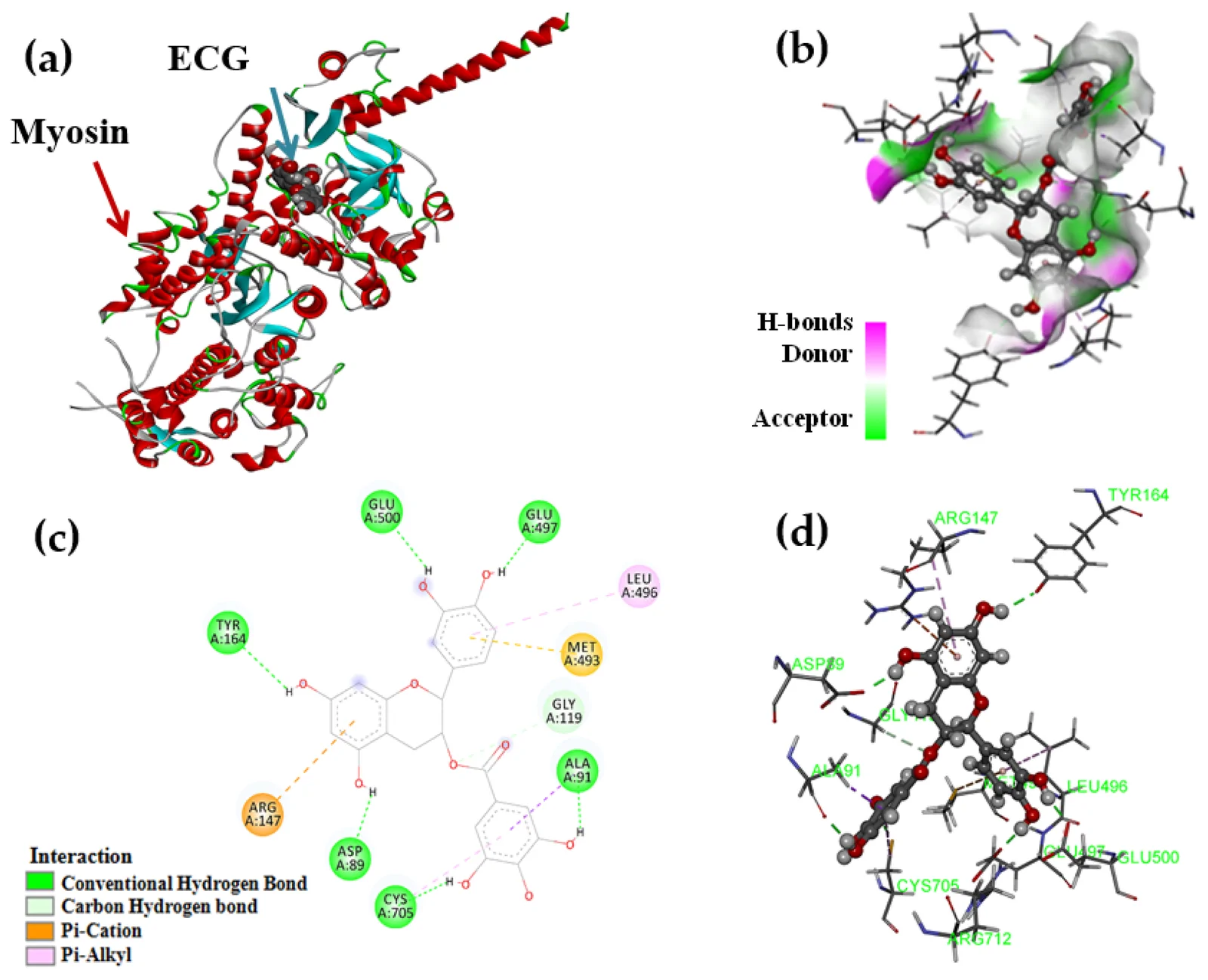
Binding modes of ECG and myosin (PDB ID: 6FSA myosin subfragment; predicted binding free energy: −9.864 kcal/mol). (**a**) The binding position of ECG on myosin; (**b**) three-dimensional binding pattern of ECG–myosin complex (green areas represent hydrogen bond acceptors, and pink areas represent hydrogen bond donors); (**c**) two-dimensional binding model of ECG and myosin; (**d**) three-dimensional binding pattern of ECG and myosin.

**Figure 16:**
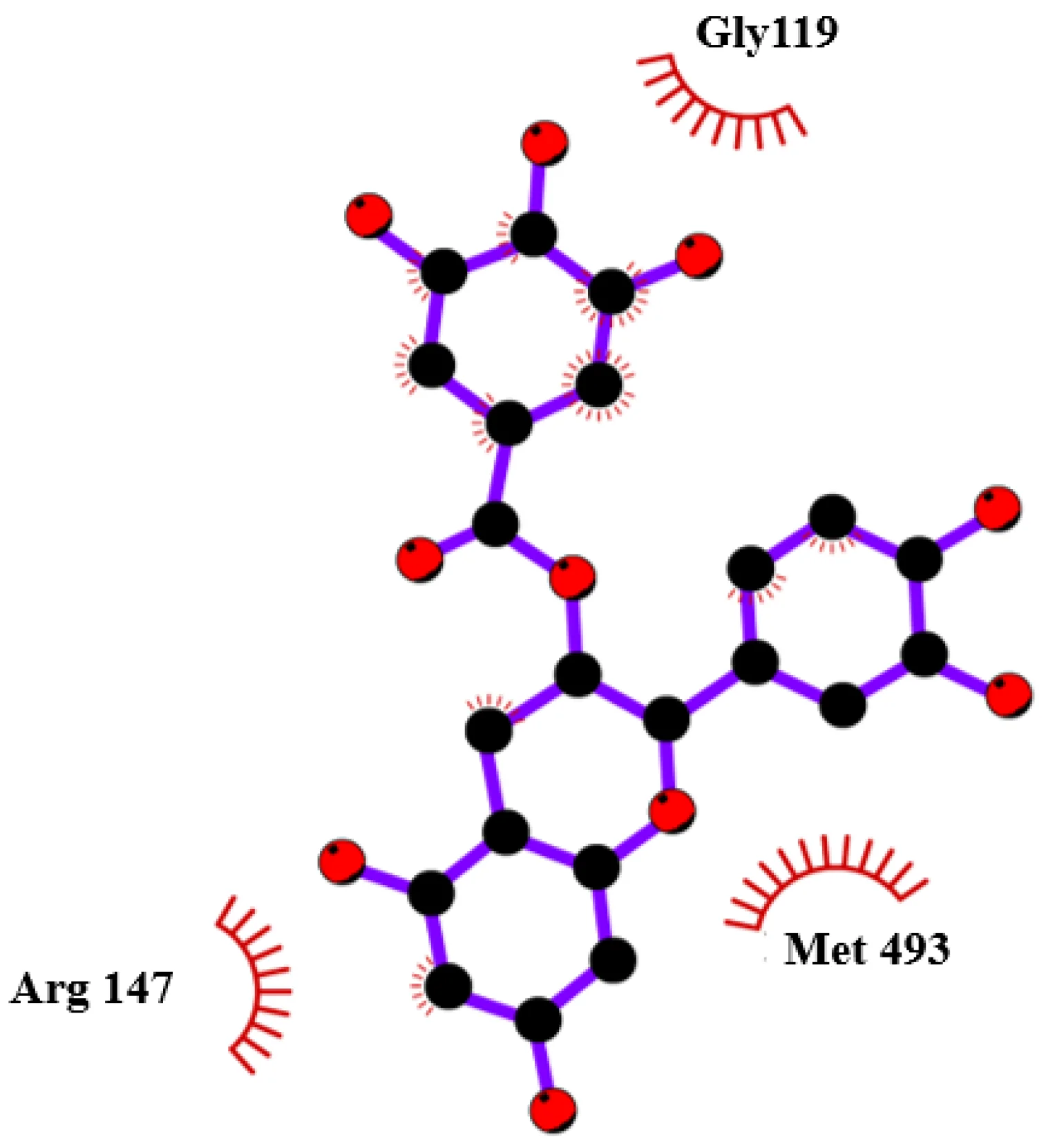
Two-dimensional binding pattern of ECG and myosin (red gear shapes indicate hydrophobic interactions).

As illustrated in Figure 15a,b, the ECG molecule was embedded inside the myosin structure and fully occupied its active binding pocket. Most interaction sites were distributed in the α-helix domain of myosin, which induced significant conformational perturbations in the α-helix structure. This may account for the ECG-induced conformational transition from α-helix to β-sheet described in Section 3.3.1. Previous studies have demonstrated that hydrogen bonds are commonly formed between the hydroxyl (–OH) groups on the aromatic rings of polyphenols and the amino (–NH_2_) moieties of proteins [36]. Consistent with these findings, Figure 15c,d and 16 visualize the detailed binding sites and intermolecular forces between ECG and myosin. The ECG molecule intensively interacts with amino acid residues lining the active pocket of myosin. Specifically, the hydroxyl groups on the B and D rings of ECG form six classical hydrogen-bond interactions with carbonyl (–C=O) and amino (–NH_2_) groups of myosin residues, including Asp89, Ala91, Glu497, Glu500, Tyr164 and Cys705. Additionally, four conjugate interaction pairs were detected between ECG and the myosin residues Leu496, Met493, Arg147 and Gly119. It is worth noting that a hydrogen bond formed between the ECG and the sulfhydryl group of Cys705 at a distance of 1.96 Å adjacent to the binding pocket, which may contribute to the stable anchoring of ECG within the myosin active pocket. It should be noted that the above results are based solely on static molecular docking and can only provide reasonable structural hypotheses regarding the binding mode of ECG with myosin and its potential sulfhydryl group protection mechanism described in Section 3.2.1. Further investigation through molecular dynamics simulations is required in the next step.

Furthermore, Figure 16 illustrates the hydrophobic interactions between ECG and myosin. The carbon atoms of ECG form three carbon–hydrogen bonds with the hydrophobic residues of myosin (Arg147, Gly119 and Met493). These hydrophobic interactions may facilitate the insertion and tight binding of ECG into the myosin active pocket, thereby enabling the formation of multiple hydrogen bonds with corresponding amino acid residues.

Overall, the molecular docking results suggest that the non-covalent interactions be- tween ECG and myosin are predominantly governed by hydrophobic interactions, with hydrogen bonds serving as auxiliary forces. This finding is well consistent with the results inferred from FTIR spectra and additive perturbation assays, as well as existing literature. Under refrigerated conditions, the binding between polyphenols and proteins is primarily mediated by synergistic non-covalent interactions, among which hydrophobic interactions and hydrogen bonds serve as the predominant driving forces. During hydrophobic binding, hydrophobic amino acid residues of proteins preferentially interact with the nonpolar aromatic rings of polyphenols [6,17,59]. Moreover, Chanphai et al. demonstrated that catechins bind to casein through hydrogen bonding and hydrophobic interactions [52].

## 4. Conclusions

This study demonstrates that ECG at concentrations of 10–40 μmol/L effectively inhibited the oxidation of bovine myosin under refrigerated storage conditions (4 °C), while exerting a distinct biphasic and dose-dependent effect on conformation and corresponding functional properties of myosin. At low concentrations (10–30 μmol/L), ECG markedly enhanced the radical scavenging ability of ECG–myosin mixtures and alleviated the oxidation of amino acid side chains. From the perspective of molecular interactions, this study suggests that the antioxidant mechanism of ECG involves entropy-driven hydrogen bonding and hydrophobic interactions, which may enable spontaneous binding between ECG and myosin. This binding promoted the transition from α-helix to β-sheet and reduced surface hydrophobicity, thereby inducing conformational rearrangement of protein secondary and tertiary structures and suppressing the oxidative denaturation of myosin. In contrast, although high-dose ECG (40 μmol/L) still possessed considerable antioxidant activity, excessive ECG aggravated conformational perturbation and structural disorder at both secondary and tertiary levels, destroyed the microstructure of myosin and increased surface hydrophobicity, ultimately reducing its structural stability.

These findings elucidate the interactions of the myosin–polyphenol system and putative mechanisms of ECG against myosin oxidation, suggesting that optimal dosage and concentration are critical for applying ECG to improve the structural and functional properties of meat proteins. It can be seen that in the application of antioxidants, the dose–response relationship is of great significance.

Further verification of ECG-mediated myosin conformational transitions and residue protective effects will be performed via molecular dynamics simulations in future work. In addition, the influences of other major components of LSPC on the oxidative stability, structural characteristics and functional properties of myosin will be systematically explored. In summary, this work provides reliable experimental evidence and theoretical support for the rational development of protein–polyphenol functional composites, and offers valuable references for the potential application of ECG and LSPC in meat processing and the development of healthy meat products in the future.

## Data Availability

The original contributions presented in this study are included in the article/Supplementary Material. Further inquiries can be directed to the corresponding author.

## Supplementary Materials

The following supporting information can be downloaded at: https://www.mdpi.com/article/10.3390/foods15183332/s1, Figure S1. The HPLC chromatogram (A) and LC-ESI-MS spectrum (B) of LSPC at 280 nm. Note: The peaks marked by numbers in Figure A represent the LSPC components were as follow: 1: Procyanidine B1 (PB1); 2: Procyanidine B3 (PB3); 3: Catechin; 4: Procyanidine B2 (PB2); 5: Procy-anidine B2 (PB2); 6: Procyanidine B6 (PB6); 7: Procyanidine B8 (PB8); 8: Procyanidine B2-gallate; 9: Procyanidine B7 (PB7); 10: Epicatechin gallate (ECG); Figure S2. Comparison of antioxidant capacity of LSPC and its major components of LSPC (A: IC50 of scavenging ·OH; B: IC 50 of scavenging the ABTS+·; C: IC50 of scavenging DPPH free radical; D: FRAP). Note: Different letters represent significant differences (*p* < 0.05) between different LSPC components; Figure S3. Antibacterial effects of important mono-case compounds of LSPC against *E. coli* (12 h) (Wang, 2021) [49]; Figure S4. SDS-PAGE electrophoreto-grams of myosin from different treatment groups. Note: Lane f, freshly extracted beef myosin; Lane a, beef myosin refrigerated for 12 h after extraction. Table S1. The composition of LSPC; Table S2. The averaged binding free energies of the simulated protein and LSPC components; Table S3. The IC50 values regarding the main components of LSPC against *E. coli* (Wang, 2021) [49]; Table S4. Thermodynamic parameters of ECG–myosin binding determined by ITC at 4 °C.

## Abbreviations

The following abbreviations are used in this manuscript:

ABTS	2,2′-amino-bis(3-ethylbenzothiazol-6-sulfonic acid) diammonium salt
ANS	1-anilino naphthalene-8-sulfonic acid
ATP	Adenosine triphosphate
ECG	Epicatechin gallate
EGTA	Ethylene Glycol Tetraacetic Acid
CD	Circular Dichroism
DPPH	1,1-diphenyl-2-trinitrophenylhydrazine
ITC	Isothermal titration calorimetry
LSPC	Lotus seedpod proanthocyanidins
PBS	Phosphate-buffered saline
