# Supplementary material for: Interaction Mechanism of Bovine Myosin Oxidation Inhibition by Epicatechin Gallate: Insights from Molecular Docking and Thermodynamic Analysis
Source: Foods. 2026 Sep 20;15(18):3332. doi: 10.3390/foods15183332 (PMC13606403; doi:10.3390/foods15183332)
Supplement: Supplementary file 1 [file foods-15-03332-s001.zip › foods-4496126-Supplementary Materials.pdf]

Interaction mechanism of bovine myosin oxidation inhibition by epicatechin gallate: Insights from molecular docking and thermodynamic analysis

of bovine myosin oxidation inhibition by epicatechin gallate: Insights from molecular docking and thermodynamic analysis

Xin Li [1], Meijuan Xu [1], Min Li [1], Xinming Yin[1], Huang Zhang [1], Xueqin Gao [1], Changbin Li [1], Zhida Sun [2], Bijun Xie [2], Jian Zou [1]*

1 *College of Food Science, Henan University of Animal Husbandry and Economy, Zhengzhou 450000, China; lixinxin_619@126.com (X.L.); Xumeijuan113@163.com (M.X.); 261016@hnuahe.edu.cn (M.L.); xinmingyin@hotmail.com (X.Y.); 81870@hnuahe.edu.cn (H.Z); kfgxq03@163.com (G.X.); lichangbin911@163.com (B.L.); zoujianzz@126.com (J.Z.)*

2 *College of Food Science and Technology, Huazhong agricultural university, Wuhan 430070, China; sunzhida@mail.hzau.edu.cn (Z.S); bijunxie@sina.com (B.X.)*

* ***Correspondence:*** *lixinxin_619@126.com (X.L.); zoujianzz@126.com (J.Z.)*

1 The components of Lotus seed pod proanthocyanidins (LSPC)

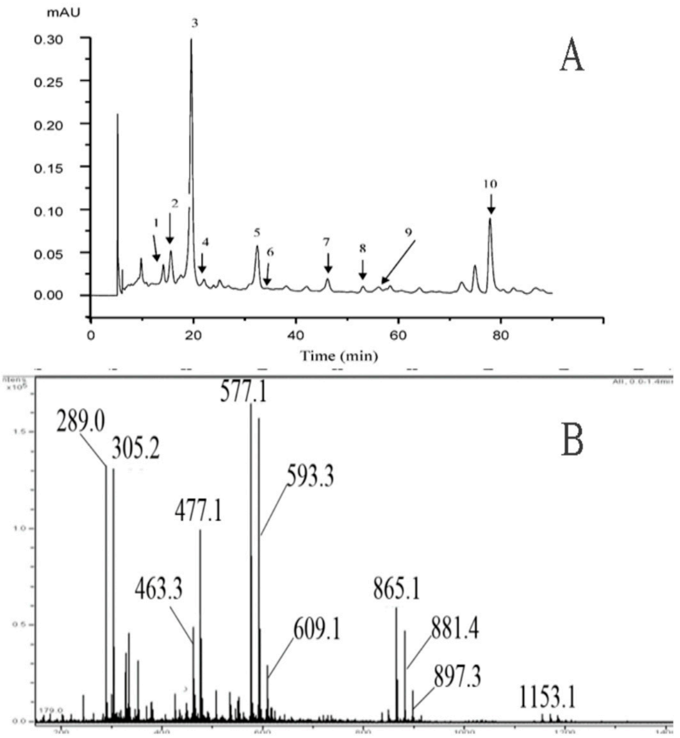

Figure S1. The HPLC chromatogram (A) and LC-ESI-MS spectrum (B) of LSPC at 280 nm. Note: The peaks marked by numbers in Figure A represent the LSPC components were as follow: 1: Procyanidine B1 (PB1); 2: Procyanidine B3 (PB3); 3: Catechin; 4: Procyanidine B2 (PB2); 5: Procyanidine B2 (PB2); 6: Procyanidine B6 (PB6); 7: Procyanidine B8 (PB8); 8: Procyanidine B2-gallate; 9: Procyanidine B7 (PB7); 10: Epicatechin gallate (ECG).

Table S1. The composition of LSPC.

Peak sequence	1	2	3	4	5	6	7	8	9	10
LSPC components	**PB1**	PB3	**Catechin**	PB4	**PB2**	PB6	PB8	PB2-gallate	PB7	**ECG**
Components(%)	**11.43**	4.50	**28.38**	2.16	**20.57**	0.05	2.25	2.34	1.76	**26.56**

2 Screening of ECG for experimental investigation

Considering antioxidant activity and binding affinity toward myosin. together, in order to objectively screen the major component from the four major LSPC constituents (C, ECG, PB1 and PB2) as the experimental object of this study, a weighted scoring strategy was established in this study. That is **the binding energy between each**

component and myosin accounted for 50% of the total weight, while antioxidant indicators occupied the remaining 50% (DPPH radical scavenging IC_{50}, hydroxyl radical scavenging IC_{50}, ABTS cation radical scavenging IC_{50}, and ferric reducing antioxidant power were equally assigned 12.5% each). Subsequently, the comprehensive weighted score of each component was calculated: ECG (0.9237) > PB2 (0.4443) > PB1 (0.3933) > C (0.1740). ECG obtained the highest comprehensive score.

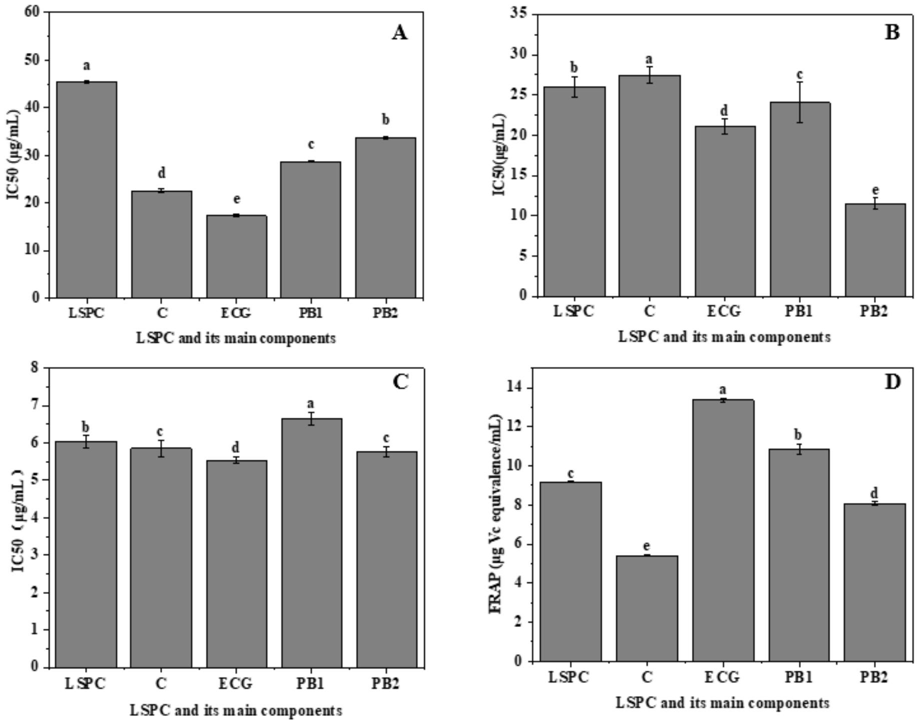

Figure S2. Comparison of antioxidant capacity of LSPC and its major components of LSPC (A: IC50 of scavenging ·OH; B: IC 50 of scavenging the $ABTS^{+\cdot}$; C: IC50 of scavenging DPPH free radical; D: FRAP).

Note: Different letters represent significant differences ($p < 0.05$) between different LSPC components.

Table S2. The averaged binding free energies of the simulated protein and LSPC components.

LSPC	ECG	C	EC	PB1	PB2
Binding free	-9.864	-7.748	-8.019	-8.886	-8.733

Note: The values in Table S1 are from the best pose.

In addition, beyond antioxidant capacity and molecular binding behavior, other functional properties of ECG were also fully considered in the screening process. Antibacterial performance is a critical indicator for the preservation of beef products. Our team's previous study has systematically compared the antibacterial activities of major LSPC components, the results were shown in Figure S3 and Table S2.

The results indicated that the monomeric components (C, EC, and ECG) exerted stronger inhibitory effects on the growth of *Escherichia coli* than polymeric dimers, suggesting that an increase in the degree of polymerization weakens the antibacterial activity of LSPC. Among the effective monomeric catechins, ester-type ECG exhibited the optimal antibacterial efficacy.

Furthermore, epigallocatechin gallate (EGCG) was used as a reference control in this work. Under consistent experimental conditions, Structurally, EGCG and ECG both contain galloyl groups, and both exhibited significantly stronger antibacterial activity than non-esterified catechins (C and EC), further verifying the key role of the galloyl group in mediating antibacterial activity. This is also one of the reasons why we chose ECG as the major component for studying LSPC.

To sum up, ECG was therefore selected as the major component of LSPC for the experiment.

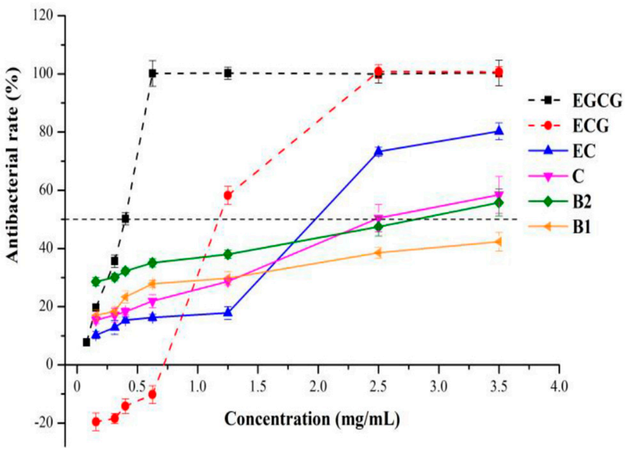

Figure S3 Antibacterial effects of important monocase compounds of LSPC against *E. coli* (12 h) (Wang, 2021) [49].

Table S3. The IC50 values regarding the main components of LSPC against *E. coli* (Wang，2021) [49].

LSPC components	**ECG**	EC	**C**	**PB2**	**PB1**
Proportion (%)	**1.18**	**1.98**	**2.51**	2.84	**--**[a]

Note: [a] IC50 value not detected

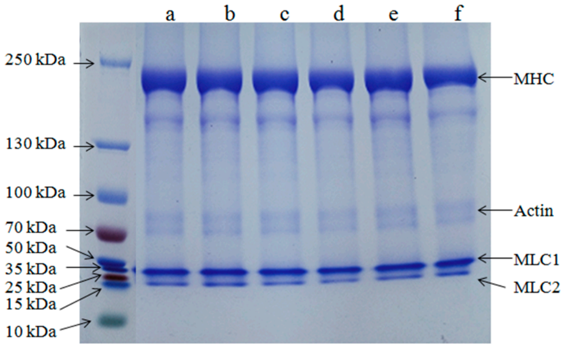

Figure S4. SDS-PAGE electrophoretograms of myosin from different treatment groups.

Note: Lane f, freshly-extracted beef myosin; Lane a, beef myosin refrigerated for 12 h after extraction.

3 ITC supplementary date

Table S4. Thermodynamic parameters of ECG–myosin binding determined by ITC at 4 °C.

Parameter	The first batch of sample			The second batch of sample			The third batch of sample		
	Value	95% CI	RSS (μJ²)	Value	95% CI	RSS (μJ²)	Value	95% CI	RSS (μJ²)
ΔH (kJ/mol)	90.17	±5.02	259.72	97.8	±4.68	233.84	100	±5.51	320.77
ΔS (J/mol·K)	464.4	\		489.9	\		497.2	\	
ΔG (kJ/mol)	-38.47	\		-37.98	\		-37.8	\	
Ka (L/mol)	1.83×10^7	1.46×10^7-2.25×10^7		1.44×10^7	1.11×10^7-1.72×10^7		1.33×10^7	1.03×10^7-1.72×10^7	
n	1.246	±0.072		1.248	±0.068		1.441	±0.121	

Note: Parameters were obtained by fitting ITC data to the single-site binding model. Bootstrap-derived 95% CIs are reported only for primary fitted parameters (*Ka, n,* and *ΔH*). *ΔG* and *ΔS* are secondary thermodynamic quantities calculated from fitted *Ka* and *ΔH*.

Reference:

Wang, J.Y. The Interaction, Synergistic Anti-Diarrhea Effect and In Vivo/Vitro Mechanism Study of Novel Carboxymethyl Pachyman-Lotus Seedpod Procyanidins Mixture. Ph.D. Dissertation, Huazhong Agricultural University, Wuhan, China, 2021. https://doi.org/10.27158/d.cnki.ghznu.2021.001252
